# Identification of Entry Factors Involved in Hepatitis C Virus Infection Based on Host-Mimicking Short Linear Motifs

**DOI:** 10.1371/journal.pcbi.1005368

**Published:** 2017-01-27

**Authors:** Austin W. T. Chiang, Walt Y. L. Wu, Ting Wang, Ming-Jing Hwang

**Affiliations:** Institute of Biomedical Sciences, Academia Sinica, Taipei, Taiwan; University of Texas at Austin, UNITED STATES

## Abstract

Host factors that facilitate viral entry into cells can, in principle, be identified from a virus-host protein interaction network, but for most viruses information for such a network is limited. To help fill this void, we developed a bioinformatics approach and applied it to hepatitis C virus (HCV) infection, which is a current concern for global health. Using this approach, we identified short linear sequence motifs, conserved in the envelope proteins of HCV (E1/E2), that potentially can bind human proteins present on the surface of hepatocytes so as to construct an HCV (envelope)-host protein interaction network. Gene Ontology functional and KEGG pathway analyses showed that the identified host proteins are enriched in cell entry and carcinogenesis functionalities. The validity of our results is supported by much published experimental data. Our general approach should be useful when developing antiviral agents, particularly those that target virus-host interactions.

## Author Summary

Viruses recruit host proteins, called entry factors, to help gain entry to host cells. Identification of entry factors can provide targets for developing antiviral drugs. By exploring the concept that short linear peptide motifs involved in human protein-protein interactions may be mimicked by viruses to hijack certain host cellular processes and thereby assist viral infection/survival, we developed a bioinformatics strategy to computationally identify entry factors of hepatitis C virus (HCV) infection, which is a worldwide health problem. Analysis of cellular functions and biochemical pathways indicated that the human proteins we identified usually play a role in cell entry and/or carcinogenesis, and results of the analysis are generally supported by experimental studies on HCV infection, including the ~80% (15 of 19) prediction rate of known HCV hepatocyte entry factors. Because molecular mimicry is a general concept, our bioinformatics strategy is a timely approach to identify new targets for antiviral research, not only for HCV but also for other viruses.

## Introduction

The conventional approach to countering viral infections has been to develop drugs that target viral genetic material or proteins. However, two major roadblocks to this strategy exist: 1) the limited number of druggable viral proteins owing to small viral genomes, and 2) drug resistance that occurs on a relatively short time scale owing to substantial viral genomic mutation rates. To circumvent these problems, over the past decade antiviral drug development has shifted from targeting viral proteins to host proteins that interact with components of the virus [1]. For example, compounds that inhibit interactions between viral and human proteins have been identified [2], including the compound LEDGIN, which targets the interaction between HIV integrase and human transcriptional coactivator p75 [3]. Cell-based genomic and proteomic assays that screen for host targets that interact with viral proteins have also been reported [4–6]. Nevertheless, given the large amount of biological data that has been accumulated from high-throughput omics-type experiments, development of a bioinformatics-sleuthing strategy that identifies potential antiviral host targets to complement experimental screens should be of considerable merit.

Herein, we describe the development of and evaluate such a bioinformatics strategy, the premise of which is based on viral “molecular mimicry,” an ability that viruses have developed over millions of years of evolution to antagonize their hosts [7]. Specifically, regions in viral proteins apparently can mimic short amino acid sequences found in human proteins involved in normal host protein-protein interactions (PPIs), so that a virus can hijack the PPI for its own purposes, such as hijacking a cellular process(es) to create the cell context needed for infection [8]. Consistent with this viral strategy, their proteins often contain host-like SLiMs (Short Linear Motifs) allowing them to interact with complementary host proteins [9, 10]. Viral SLiMs can be identified by sequence comparison with those with the ability to bind eukaryotic protein domains as catalogued in the database ELM (Eukaryotic Linear Motif) [11]. The viral SLiMs, the host proteins that contain a matched SLiM-binding domain, and these proteins’ interacting partners in the human PPI network then form a putative virus-host interaction network, which can be integrated with known functional and network properties of cellular pathways, including those involved in disease states, thereby allowing identification of host factors whose native functions may be altered or hijacked by the virus to facilitate its infection and/or another of its life cycle stages.

To examine this molecular mimicry strategy and the feasibility of using human PPI network data to complement experimental studies, we focused on the hepatitis C virus (HCV) envelope proteins, E1 and E2, for the following reasons: First, HCV infection is a major health problem worldwide [12], and HCV E1 and E2 are known to play essential roles in HCV entry into human hepatocytes [13]; investigating E1 and E2 might therefore lead us to identify novel HCV entry factors as targets for drug design—an important step toward developing more effective anti-HCV drugs. Second, the complexity of the network and functional analysis required was significantly reduced because only liver cell surface proteins of the human proteome need to be considered. Third, many HCV entry–facilitating human proteins have been identified, which allowed us to compare *in silico* predictions with published experimental data.

**Fig 1:**
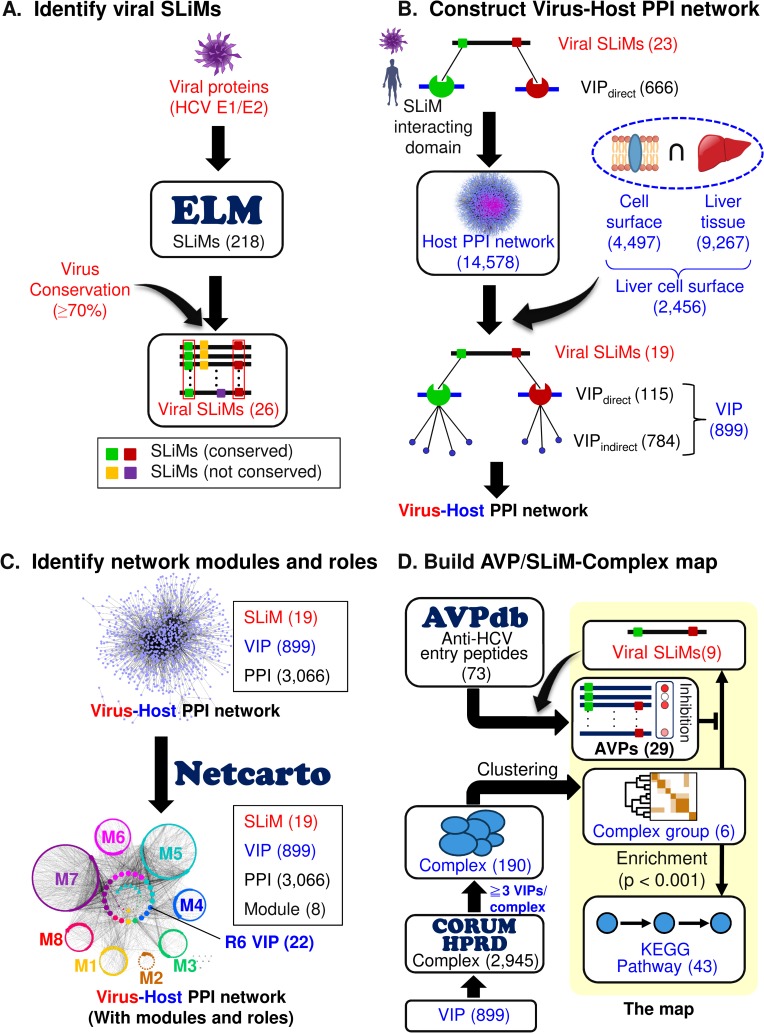
The four components of our bioinformatics strategy. **(A)** Identify human-type short linear motifs (SLiMs) found in a viral protein(s). **(B)** Construct a virus-host-PPI network. **(C)** Identify network modules and roles of the network nodes. **(D)** Build a map of AVP/SLiM-protein complexes. In parentheses are the numbers of motifs, proteins, modules, pathways, etc., identified in this study (see Methods for details).

Using the HCV E1 and E2 sequences as examples, Fig 1 schematically depicts the four main components of our approach, which are detailed in Methods. First, conserved E1/E2 sequences from various HCV strains are identified that correspond to SLiMs found in the eukaryotic linear motif (ELM) database (Fig 1A). Next, proteins on the surface of human hepatocytes known to bind such SLiMs are identified (they are called VIPs_direct_ for Virus-Interacting host Proteins), as are host proteins (VIPs_indirect_) that bind VIPs_direct_ (Fig 1B). Taking the experimentally determined interactions between VIPs_direct_ and VIPs_indirect_ from the original human PPI network ([14]; see Methods), a virus-host PPI network is then extracted that is constructed of the viral SLiMs and human VIPs (Fig 1B and 1C). This network contains modules (communities) of functionally related host proteins (nodes) and links (PPIs) within and between the modules connecting interacting nodes (Fig 1C). Finally, a map connecting SLiMs to known antiviral peptides (AVPs) and complexes containing multiple (≥3) SLiM-interacting proteins is produced (Fig 1D), for which statistical analyses are carried out to find enriched functionalities and pathways that correlate with published experimental data.

As shown below, the results show that the proteins we identified as possible hepatitis C virus (HCV) entry factors: 1) have a statistically significant propensity to be found in the PHISTO and EHCO lists, which contain experimentally identified HCV-interacting proteins and genes differentially expressed in HCV-induced hepatocellular carcinoma, respectively [15, 16]; 2) have greater coverage of known HCV entry factors than a functional genomics screening experiment [5]; and 3) contain domains that can bind short linear motifs that are also present in many antiviral peptides with experimentally demonstrated activities against HCV infection. These results suggest that, to eliminate viral infection, more attention should be paid to sequence motifs involved in host protein-protein interactions because these motifs may be subject to molecular mimicry by viruses.

## Results

### A SLiM-derived HCV-human PPI network

We identified 19 SLiMs on HCV E1 and E2 that might bind various human protein domains (Fig 1B). Screening for human hepatocyte surface proteins in conjunction with the available human PPI network yielded 115 VIPs_direct_ containing at least one SLiM-binding domain. These proteins and their VIPs_indirect_, which interact with them, constitute a subset of the experimentally derived human PPI network [14] that might be directly or indirectly influenced by the mimicking HCV SLiMs. It follows, according to the premise of our molecular mimicry strategy, that the host VIPs_direct_ and VIPs_indirect_ potentially facilitate or inhibit HCV entry and, along with the viral SLiMs, they formed a viral-host PPI network (Fig 1B and 1C).

### Functional modules and network roles

**Fig 2:**
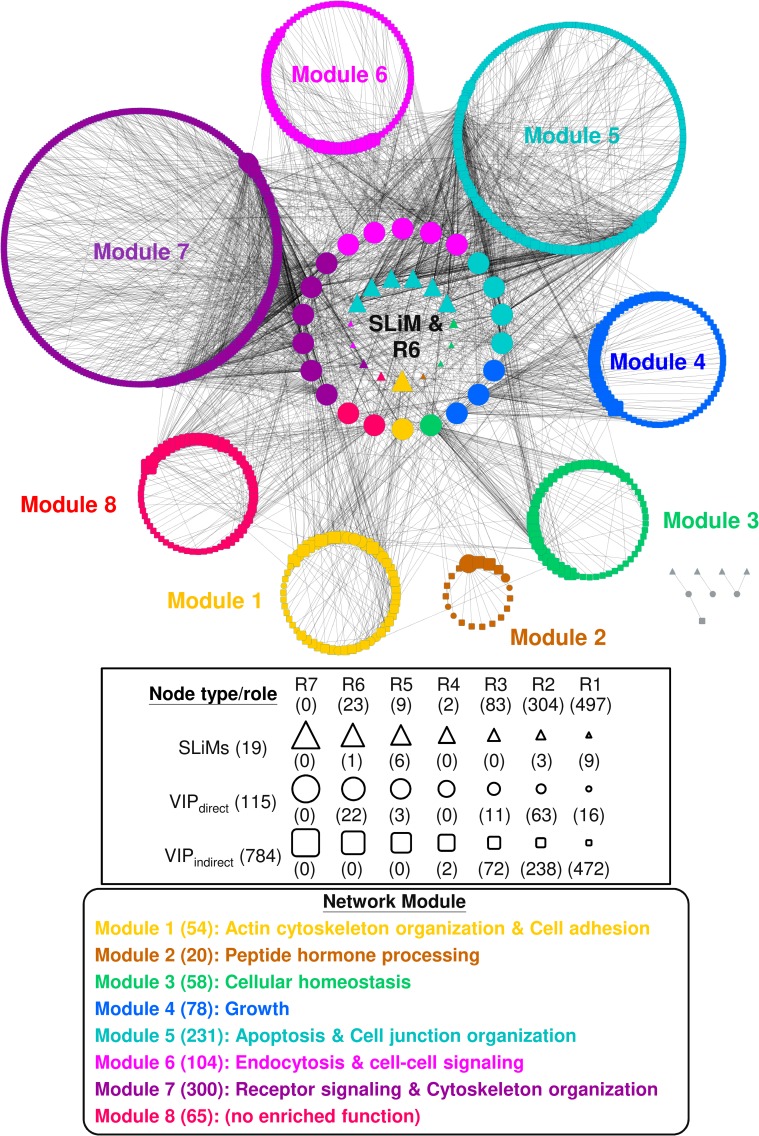
Modules and roles of the nodes of the HCV-Human PPI network. The network was produced using the procedures described in Fig 1A–1C, with the modules and the roles of network nodes determined by NetCarto (see Methods). The three types of network nodes are represented as triangles for SLiMs; circles for VIPs_direct_; and squares for VIPs_indirect_. Their network roles (R7 to R1) are depicted as symbols of decreasing size. All nodes are color-coded according to their module. A representative function(s) of each module was derived from an enrichment analysis of GO terms associated with its nodes followed by a summary of Revigo representatives [21] (see Methods and S1 Table). Colored in gray in the lower right corner are eight isolated nodes.

**Fig 3:**
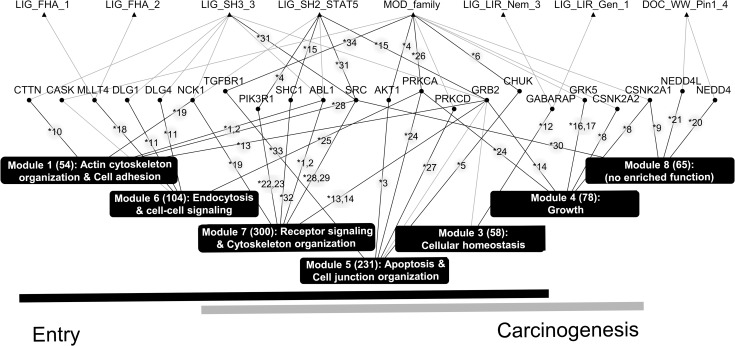
Relationships between SLiMs, R6 VIPs, and network modules. Of the 19 SLiMs identified for HCV E1 and E2 in Fig 1C, 13 (including six grouped in the MOD_family; top row) are directly connected to one or more of the 22 R6 VIPs_direct_ (middle row) in the virus-host PPI network (Fig 2). The MOD_family contains MOD_CK1_1, MOD_CK2_1, MOD_GSK3_1, MOD_NEK2_1, MOD_NEK2_2, and MOD_ProDKin_1; all are targets of a kinase. An R6 VIP_direct_ and a module(s) (bottom, boxed in black) are considered to be connected if more than 10% of the interacting partners of the VIP_direct_ belong to the module. Based on this criterion, module 2 is not connected to an R6 VIP_direct_ and, therefore, is not included in the figure. The validity of the connections displayed as solid dark lines is supported by published experimental evidence. The corresponding reference number(s) (indicated by an asterisk) are provided in S3 Table. The dark and light horizontal bars at the bottom of the figure identify modules with the functionality of *entry* and/or *carcinogenesis*, respectively.

Given a network, algorithms are available to extract network properties [17]. Using NetCarto, a tool for network module discovery [18], we found that the resulting viral-host PPI network for HCV infection is organized into eight modules with 23 R6 (global connector) hubs (Fig 2). A global connector hub (R6) is defined as a node with many links to most of the other network modules (see Methods for definitions on roles of network nodes), and as such it is thought to play an important role in connecting different functional modules. Consistent with the definition, whereas most of the 115 VIPs_direct_ interact with only a few other host proteins, these 22 R6 hub proteins (the twenty-third R6 hub is a viral SLiM) have many interaction partners. This may imply that these R6 hubs could be important host factors for HCV infection, and could serve as targets for designing anti-HCV drugs (see AVPs analysis below). Further analysis showed that 15 out of the 22 R6 hubs (P < 2.2 × 10^−16^; S1 Fig) were also hub proteins in the experimentally derived PPI network of human liver cell surface proteins, suggesting that most of these VIPs_direct_ R6 hubs have important functions for the host, irrespective of HCV infection. This is in line with the finding that viruses tend to target host hub proteins for perturbing key pathways (or biological processes) to benefit viral infections [19]. Interestingly, Gene Ontology (GO) enrichment analysis [20] and Revigo summarization [21] of the enriched GO annotations (S1 Table) revealed that the representative functions of seven of the eight modules belong to one, or both, of the two main functionalities: *entry* and *carcinogenesis* (Fig 3 and S2 Table).

As described below, much experimental data is available to support our *in silico* observations. For example, “*Cytoskeleton organization*” (modules 1 and 7; Figs 2 and 3) is an essential cellular process that allows HCV to migrate to the tight junction where internalization and endocytosis of the virion occur [22]. Notably, some of the proteins of the R6 hubs are involved in this cellular process. According to our hypothesis, cellular processes involved in “*Cytoskeleton organization*” might be hijacked by HCV if one or more of its E1/E2 SLiMs can bind at least one of the following six R6 proteins (in this work we use official gene symbols to represent proteins encoded by the corresponding genes): PIK3R1, which enhances actin reorganization by activating PI3K-AKT signaling [23]; SRC, which induces changes in the cytoskeleton by binding and activating FAK [24]; and ABL1 [25], GRB2 [26], NCK1 [27], and CTTN [28], proteins which regulate cytoskeleton rearrangement.

A function found for module 5 is “*apoptosis*” (Figs 2 and 3), which is an essential cellular process as it prevents HCV from spreading in the host by inducing the death of HCV-infected cells [29]. However, E2 can suppress cellular apoptosis resulting in HCV survival [30]. In our viral-host PPI network, there are five module 5-associated R6 proteins, four of them, AKT1 [23, 31], CHUK [32, 33], PRKCA [34, 35], and TGFBR1 [36, 37] have a role in apoptosis and their activities and/or expressions are known to be affected by HCV infection, although the specific effects of E1 and/or E2 on CHUK, PRKCA, and TGFBR1 activities have yet to be determined. The apoptosis-regulating role of the fifth R6 protein PRKCD [38] during HCV infection has also not been examined.

Another representative function of module 7, “*receptor signaling*,” contributes to HCV entry [39] and carcinogenesis [40]. Specifically, HCV infection triggers EGFR signaling and stimulates its downstream signaling, including those of HRAS and PI3K-AKT [39]. Activation of these pathways enhances HCV entry [23, 39] and increases the proliferation of hepatocytes, which may contribute to hepatocellular carcinogenesis [40]. Three R6 proteins are associated with receptor signaling: PIK3R1, a PI3K subunit and a crucial participant in PI3K-AKT signaling [41], and GRB2 [42] and SHC1 [43], two key adaptor proteins of EGFR signaling, which when silenced substantially impair HCV entry [39].

Additional published experimental data that support the relationships between the R6 proteins and the functions of the viral-host PPI network modules are summarized in S3 Table.

### VIPs also found in PHISTO and EHCO

We identified 899 VIPs using our scheme. To evaluate the validity of our findings, we compared our list of VIPs with those in the PHISTO (Pathogen-Host Interaction Search Tool) dataset, which contains a list of experimentally verified HCV-interacting human proteins [16]. There are a total of 698 HCV-interacting human proteins in PHISTO, of which 160 are in the set of 2,456 liver cell surface proteins (Fig 1B). Of the 160 HCV-interacting hepatocyte surface proteins, 158 are annotated specifically as interacting with the polyprotein of HCV (S4 Table), which contains E1 and E2. As shown in S2A Fig, the 899 VIPs tended to contain members from the 158 subset of the PHISTO list, with 92 proteins overlapped between the two (P = 9.2 × 10^−9^; S2A Fig). Similarly, the predicted interactions between HCV(SLiMs) and VIPs, i.e. the edges of the viral-host PPI network, were enriched in PHISTO (P = 4.3 × 10^−4^ and 9.6 × 10^−5^ for direct and indirect interactions, respectively; see S3 Fig and S4 Table). These results indicate that our bioinformatics approach preferentially identified HCV-interacting human proteins.

Four of the identified virus-host PPI network modules are associated with carcinogenic processes (Fig 3), which is a somewhat unexpected result as E1 and E2 are usually only considered to be entry proteins [13]; however, this association is consistent with known oncogenic effects of E1 and E2 [44, 45]. To further evaluate the involvement of the VIPs in hepatocellular carcinoma (HCC), we assessed if a significant number of those VIPs are found in the EHCO (Encyclopedia of Hepatocellular Carcinoma genes Online) [15] dataset. Of the 614 genes that are differentially expressed in HCV-caused HCC in EHCO, 194 are expressed on liver cell surface, and 91 of their encoded proteins were identified as VIPs (P = 1.4 × 10^−3^; S2B Fig), further supporting the notion that E1/E2 interact with host proteins that have a role in carcinogenesis.

### Comparison with a siRNA-screening experiment list

Recently, a set of host factors for HCV entry was identified in a large-scale siRNA screening experiment reported by Li and coworkers [5]. However, the authors of that study identified only four of the 15 human proteins found on hepatocyte surface and known to be associated with HCV entry (Table 1). By comparison, we identified 11 of these entry factors known before Li and colleagues performed their study, and we also identified the four proteins found by them (Table 1). Three of the known entry factors that our study did not find, CLDN1, SCARB1, and CD209, are connected to at least one VIP_indirect_ in the human PPI network, but are not VIPs themselves (S4A–S4C Fig). The fourth, NPC1L1, which we did not identify, lacks information of interaction with any of the VIPs in the network (S4D Fig).

**Table 1:** Comparison with known entry factors of HCV infection.

Gene symbol	Protein name	Identified ^a^		
		This study (15)	Li_2014^b^ (8)	Literature^c^ (15)
CD81	CD81 antigen	Y	Y	Y
CDC42	CDC42	Y	Y	Y
RAC1	Ras related C3 botulinum toxin substrate 1	Y	Y	Y
APOE	Apolipoprotein E	Y	N	Y
EGFR	EGF receptor	Y	N	Y
EPHA2	EphA2	Y	N	Y
HRAS	H-Ras	Y	N	Y
LDLR	Low density lipoprotein receptor	Y	N	Y
OCLN	Occludin	Y	N	Y
PCSK9	Proprotein convertase PC9	Y	N	Y
TFRC	Transferrin receptor	Y	N	Y
CLDN1	Claudin 1	N	Y	Y
CD209	DC-SIGN	N	N	Y
NPC1L1	NPC1 like 1	N	N	Y
SCARB1	Scavenger receptor class B member 1	N	N	Y
ARHGEF7	Rho guanine nucleotide exchange factor 7	Y	Y	N
CDH1	Cadherin-1 precursor	Y	Y	N
RAB34	Ras-related protein Rab-34, isoform NARR	Y	Y	N
RBP4	Retinol-binding protein 4 precursor	Y	Y	N

^a^ Y, yes; N, no.

^b^Eight proteins (ARRDC2, CHKA, CYBA, DDX3X, FASN, MAP4, PIK4CA, and ROCK2) identified in [5] as HCV entry factors are not expressed on the liver cell surface according to the HPRD [46] and the Human Proteinpedia [47]; thus, these factors were excluded from our analysis.

^c^Proteins annotated with “Entry” and “Attachment” in the column of “HCV Life Cycle Stages Affected” in S9 Table in [5] were compared, however CLEC4M, FASN, IFITM1, PIK4CA, ROCK2, and SDC1 in S9 Table of the report were not included because they were not expressed on the liver cell surface according to the HPRD [46] and the Human Proteinpedia [47].

CD81, OCLN, CLDN1 and SCARB1 are arguably the four best known HCV entry-related factors [48]. Of them, we identified CD81 and OCLN as VIPs_indirect_ but failed to find CLDN1 and SCARB1 as noted above. Interestingly, transgenic expression of human CD81 and OCLN in mouse enabled HCV infection of mouse hepatocytes, whereas transgenic expression of human CLDN1 or SCARB1 was not necessary for mouse cell to be infected by HCV [49]. As a VIP_indirect_, CD81 is not predicted to possess a domain that can directly bind an E1/E2 SLiM, which might be considered to contradict a report suggesting a physical interaction between HCV E2 and CD81 [50] can occur. Nonetheless, viruses employ other strategies to interact with host proteins [7], and indeed, substitution mutation experiments suggested that HCV E2 uses a non-sequential motif to bind CD81 [51], which we would not have uncovered using the SLiM-based approach. Altogether, our approach found more known HCV hepatocyte entry host factors (15 of 19 (79%); Table 1) than did the siRNA functional genomics-screening assay of Li and colleagues. Furthermore, an ROC (Receiver Operating Characteristic [52]) curve analysis accounting for not only sensitivity but also specificity showed that the *in silico* predictions yielded an AUC (area under curve) of 0.68, which is almost as good as that (0.70) of Li et al.’s experimental screening (S5 Fig). Note that including protein complexes in the analysis (see below) significantly improved on specificity for the *in silico* predictions while maintaining the overall performance; similarly, in Li et al.’s experiment, a large number of genes (19,277) had been removed by a genome-wide genetic screen [53] prior to the siRNA functional analysis [5], and these were not included in the ROC curve analysis, giving rise to a much higher specificity for Li et al.’s experiment (S5 Fig). In addition, because all of those we predicted as novel ones were treated as false positives in these calculations, the actual performance of our predictions could possibly be better.

### Protein complexes and KEGG pathways

Viruses are known to target protein complexes so as to heavily perturb host functions and induce disease states, e.g., carcinogenesis [6, 54]. It is, therefore, of interest to identify human protein complexes that might be perturbed by HCV infection, including those involving VIPs_direct_ and VIPs_indirect_. Knowledge of such protein complexes would also greatly reduce the number of VIPs needed to be considered for pathway analysis and experimentation.

**Fig 4:**
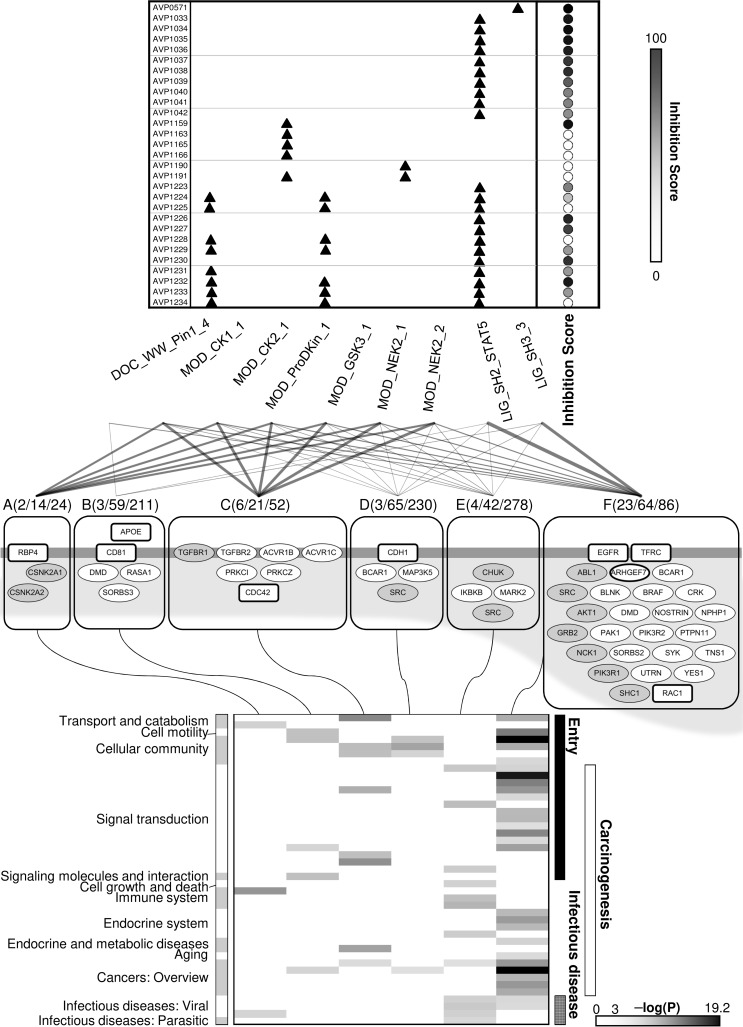
The AVP/SLiM-protein complex map. Top panel: (left column) a list of AVPs with their AVPdb identification numbers that have an amino acid sequence containing one or more HCV E1/E2 SLiMs (indicated by the triangles and named at the bottom of the panel) that can bind to a subunit of a protein complex belonging to one of the six main complex groups. The relative efficacies of these AVPs in inhibiting HCV entry according to data provided in the AVPdb [56] are indicated by the circles column in the panel with the shading score shown to the right of the panel. Middle panel: the network connecting the nine SLiMs to their group A-F complex target(s). Within each group are VIPs of all the complexes: ovals represent VIPs_direct_ (gray ovals are R6 VIPs_direct_), and rectangles represent VIPs_indirect_ (not all VIPs_indirect_ are shown). The ovals and rectangles in bold outline are known HCV entry factors. The horizontal bar represents the plasma membrane, the VIPs below the bar and within the shaded area are ‘peripheral membrane proteins’, and those spanning the bar are ‘integral membrane proteins’. APOE is a peripheral membrane protein located at the extracellular side of the cell membrane. A connection between a viral SLiM and a complex group indicates that at least one protein in the group is targeted by HCV E1 and/or E2 via the viral SLiM. The thickness of the connection roughly scales to the number of proteins targeted by the SLiM in the group. The three numbers in the parenthesis are the number of VIPs_direct_, total VIPs (i.e., VIPs_direct_ plus VIPs_indirect_), and unique subunits in the complex group. Bottom panel: the KEGG pathways enriched in the complex group (Benjamini-Hochberg adjusted P < 0.001) (see S6 Table for the pathway names). The gray scale at bottom right indicate the significance of the P-values. The functions of the individual KEGG pathways are shown at the left of the panel and the main functionality at the right of the panel.

By mapping the VIPs to protein complexes in the HPRD [46] and CORUM [55] databases (these databases categorize human and mammalian protein complexes, respectively) and clustering according to shared subunits, we identified six groups of protein complexes (Fig 4 and S6 Fig and S5 Table) that HCV may target by interaction with the VIPs of the complexes. These six groups contain 231 of the 899 VIPs and nine of the 19 viral SLiMs, with six of the nine SLiMs capable of binding the same protein domains (Fig 4). A comparison of our results with those obtained using the same mapping procedure and the same number of randomly selected proteins showed that the tendency of the HCV SLiMs-derived VIPs to be present in these protein complexes is unlikely to occur by chance (P < 1.0 × 10^−5^; S7 Fig). Furthermore, 11 of the 22 R6 hub proteins are present in these HCV-targeted complexes, (P = 1.1 × 10^−2^; S2C Fig). Moreover, these 231 VIPs are significantly enriched by members of the PHISTO, EHCO, and known HCV entry factor lists (P = 1.5 × 10^−11^, S2D Fig; P = 1.8 × 10^−7^, S2E Fig; and P = 4.8 × 10^−3^, S2F Fig; respectively).

Two known HCV entry factors, APOE and HRAS, and a novel entry factor ITGB identified by Zona and colleagues [39] are components of a single complex with CD81 [39, 57]. Of this CD81-complex, we identified several subunits, ADAM10, APOE, CD59, CD9, HRAS, ITGB1 and SCAM, as VIPs, this complex hence meets our criterion of ≥3 VIPs for an HCV-targeted host protein complex even though information of this complex was not used in our analysis because the latest compilations of CORUM and HPRD, in 2012 and 2009, respectively, predate the work of Zona and colleagues. This example therefore illustrates the merit of our approach in general, and the inclusion of protein complexes in the analysis in particular.

As detailed in the S1 Appendix, there are three main functionalities associated with KEGG pathways that are enriched in the HCV-targeted protein complexes: *entry*, *carcinogenesis*, and *infectious disease*; the first two overlap substantially, as is shown by the analysis of the GO enrichment terms associated with the network modules (Fig 3). Examination of the KEGG pathways enriched in complex-forming VIPs helps us to understand the roles the complexes might play during HCV infection. For example, *TGF-beta signaling* (P = 1.2 × 10^−6^), *Endocytosis* (P = 4.8 × 10^−11^), and *Adherens junction* (P = 7.6 × 10^−7^) are among KEGG pathways enriched in group C complexes (S6 Table) in which the VIPs_direct_ TGFBR1, TGFBR2, ACVR1B, and ACVR1C are signaling receptors [58] and PRKCZ and PRKCI are protein kinases involved in endocytosis and adherens junction remodeling [59]. Our analysis revealed that several of the enriched pathways are involved in more than one step of HCV entry, and that the envelope proteins may regulate immune responses to HCV infection, affect hormone-related signaling pathways, and modulate HCC progression. These suggestions are in line with many experimental studies (S1 Appendix), including the report that HCV E1 and E2 can alter *RIG-I-like receptor signaling* and *Toll-like receptor signaling* [60]. Finally, the presence of enriched pathways associated with *infectious disease* suggests that some of the VIP-containing protein complexes may also be targets of other viruses, which is consistent with reports of coinfection of two or more different viruses (see, e.g. [61]).

However, when compared to all the SLiM-binding hepatocyte surface proteins and their binding partners in the human PPI network, the identified HCV-targeted protein complexes were shown to be significantly enriched in KEGG pathways belonging to the functionality of *cell entry* (P = 3.7 × 10^−2^) and *carcinogenesis* (P = 2.7 × 10^−2^), but not *infectious disease* (P = 3.8 × 10^−1^) (see S8 Fig). This may suggest that *infectious disease* is more likely than the other two functionalities to be influenced by many of these proteins with a domain that can bind other SLiMs of the ELM database.

### AVPs and SLiMs

Peptides that can interfere with virus-host interactions are potential antiviral drugs [62, 63]. The viral SLiMs that we identified may, therefore, be useful scaffolds upon which to build AVPs. A search of the AVPdb [56] returned 73 AVPs that have been examined for entry-related, anti-HCV infection (Fig 1D), with more than one-third (29) harboring at least one of the nine identified SLiMs that might target a VIP residing in at least one of the six main protein complex groups. A statistical test indicated that these nine SLiMs were as likely to be also present in other AVPs (P = 8.8 × 10^−1^, S9A Fig), suggesting that, besides SLiMs, other parts of the AVP sequences are required to determine entry-related anti-HCV activities. Notably, however, many of the 29 AVPs have been shown to actively suppress HCV entry in cell-based assays (Fig 4, top panel). Although the molecular mechanisms associated with the anti-HCV activities of these AVPs have not been fully elucidated, it is tempting to speculate that, because they contain an HCV SLiM, they may interfere with an HCV-host protein interaction by preferentially binding the host protein and, thereby, inhibiting HCV entry.

### Case examples

We present two examples to demonstrate how our bioinformatics procedures can be used to identify protein complexes known to play a role in HCV infection and/or pathology.

**Fig 5:**
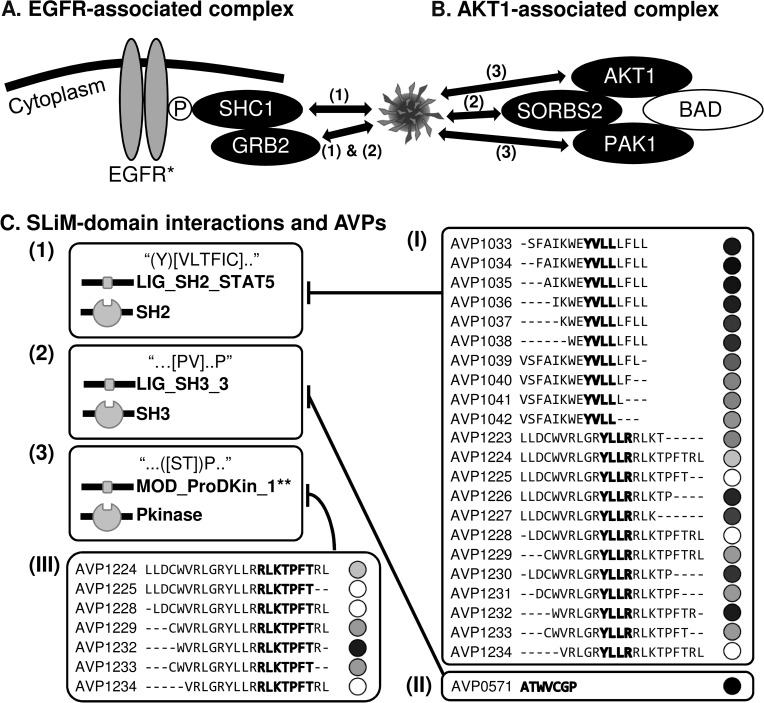
Case examples. **(A)** The EGFR-associated complex (S5 Table, Complex ID: 176). **(B)** The AKT1-associated complex (S5 Table, Complex ID: 180). **(C)** Types of SLiM-domain interactions (indicated by (1), (2), and (3)) that mediate the targeting of protein complexes, and three sets of AVPs (indicated by (I), (II) and (III)) containing the corresponding SLiM that exhibits inhibition activity (indicated by bar-headed arrows) are shown. Proteins represented by black ovals are VIPs_direct_; by gray ovals are VIPs_indirect_; and by white ovals are not VIPs (i.e. not in the virus-host PPI network). Double-headed arrows in panel A and B indicate predicted HCV-host PPIs in our analysis. Viral SLiM(s) are highlighted within the amino acid sequence of the AVP and are accompanied by its relative efficacy (in shaded circles) in inhibiting HCV entry (see the vertical bar for the normalized inhibition score in Fig 4 on the right of its top panel). The regular expression of each SLiM sequence as annotated in the ELM database [11] is shown within quotation marks in the SLiM-domain interactions (1), (2), and (3). *EGFR is a known HCV entry factor. **MOD_ProDKin_1 is representative of the MOD_family SLiMs (see Fig 3).

The first example (Fig 5A), involves the complex containing EGFR, SHC1, and GRB2 (group F complex, Fig 4). This complex (S5 Table, complex ID: 176) mediates HRAS signaling critical for HCV entry [39]. According to our results, HCV E1/E2 might interact directly with SHC1 and GRB2 (both are R6 VIPs_direct_) and indirectly with EGFR (a VIP_indirect_) via the SLiMs of LIG_SH2_STAT5 and/or LIG_SH3_3 (Fig 5A and 5C). Furthermore, of the 23 AVPs that contain the same SLiM (22 with LIG_SH2_STAT5 and one with LIG_SH3_3), 20 (86%) are shown to inhibit HCV entry (Fig 5C, I and II), and this percentage is shown to be statistically significant (P < 1.0 × 10^−4^, S9B Fig).

The second example, the AKT1-associated complex (also a group F complex, Fig 4), is presented in Fig 5B. When being a part of this complex (S5 Table, complex ID: 180), AKT1 can inhibit apoptosis induced by BAD overexpression [64], and inhibition of BAD-mediated apoptosis contributes to HCC progression [65]. Our analysis suggests that HCV E1 and/or E2 may interact with the AKT1-associated complex by targeting one or more of its members, i.e., AKT1 and PAK1 through SLiMs of the MOD_family (MOD_NEK2_2 of E1; MOD_CK1_1, MOD_CK2_1, MOD_GSK3_1, MOD_NEK2_1 of E2, and MOD_ProDKin_1 of E1 and E2), and SORBS2, an SH3 domain-containing protein, through the SLiM of LIG_SH3_3. Together with the report that HCV E2 can induce AKT phosphorylation to facilitate HCV entry [23], these results suggest that a viral SLiM and protein domain association may be involved in viral entry and virus-induced carcinogenesis. Furthermore, as with the first example, the majority (four out of seven; P < 1.0 × 10^−4^, S9C Fig) of the AVPs containing a MOD_family SLiM inhibits HCV entry (Fig 5C (III)).

## Discussion

Despite recent, rapid advances in high-throughput experiments, all characterized networks of virus-host interactions remain vastly incomplete. To fill this void, several studies have incorporated bioinformatics information [66] such as that obtained by text mining experimental reports from the literature [67]. In this work, we described a strategy, identifying eukaryote-interacting SLiMs found in viruses to find human proteins that may be “hijacked” by HCV for its entry via “molecular mimicry” of the SLiMs. With the identification of these human proteins, i.e., VIPs, we then built a virus-host PPI network by exploring the human PPI network for functions that may be modulated and/or productively used by the virus.

Per our “molecular mimicry” hypothesis, more than half of the hepatocyte surface proteins could be VIPs for a SLiM from the ELM database to interact with directly or indirectly (1,320/2,456, see S8 Fig legend), and more than one third (899/2,456, Fig 1B) for a SLiM harbored by HCV E1/E2 alone (Fig 1B). This suggests that SLiMs by themselves are of low binding specificity to protein domains, and thus most of the predicted VIPs are likely false positives. However, as demonstrated by the results presented above, by integrating with a variety of experimental data and information, especially with network and functional analysis, the number of VIPs (hence false positives) predicted can be greatly reduced and a manageable list of viable candidate proteins can be extracted to complement and guide further experimental investigations.

Our functional analysis shows that the SLiMs-derived VIPs of HCV infection and the related host protein complexes are involved in two major types of cellular functions, one associated with viral entry and the other with carcinogenesis and/or infectious disease (Figs 3 and 4). Although the inclusion of the second category was somewhat unexpected because HCV E1 and E2 were used to find SLiMs, a role for E1/E2 in carcinogenesis has been demonstrated [44, 45] and multi-functionality of other viral envelope proteins has also been documented [68–70]: For example, hemagglutinin, an envelope protein of the influenza virus, is involved in viral entry but also activates NF-κB when expressed in 293T and Hela cells [69]. Many of the predicted interactions between the SLiMs found in HCV E1/E2 and the human VIPs that occupy a prominent role (R6, global connector hub) in the virus-host PPI network, and the relationships between these R6 VIPs and their deduced functional modules, are supported by published experimental evidence (Fig 3). Together with the results from a similar approach used to predict HIV-interacting human proteins [71] and the report that virus-host interactions may be assisted by host-like SLiMs [10], evidence is mounting to support the suggestion that, via SLiMs, host proteins can be “hijacked” and host functions rewired by pathogens, a phenomenon that has been extensively reviewed at the pathway level [8]. Further supporting this premise, some HCV E1/E2 SLiMs, particularly those that might interact with major protein complexes, are found in many AVPs that inhibit HCV infection (Fig 4).

Although recently approved direct-acting antiviral (DAA) treatments have improved the virologic response rate to >90% for most types of chronic hepatitis C infections, new, hard-to-treat HCV strains including genotype 3 and DAA-resistant variants generated from DAA-treated patients, have appeared [72]. Because DAA-resistant strains are disseminated mainly through cell-to-cell transmission rather than cell-free transmission [73], the former route will be key to eradicating HCV infection. Several known HCV entry factors for cell-free transmission, e.g., EGFR, CLDN1, OCLN, and SCARB1 are also involved in cell-to-cell transmission [74–76]. Additionally, cell-to-cell transmission independent of CD81, the most well studied binding receptor for HCV E2 [13], has been reported [77–79]. Taken together and given that HCV E1/E2 are indispensable for cell-to-cell transmission of the virus [79], other host factors that can interact with these viral proteins need to be identified. Although the mechanism(s) of HCV cell-to-cell transmission is not yet understood, this type of transmission has been associated with cell adhesion molecules [80], many of which belong to the KEGG pathway category of Cellular community. Interestingly, pathways in this category are significantly enriched in four of the six protein complex groups (all except group A and E complexes; Fig 4), suggesting that some of the complex-associated VIPs may be a good starting point to study cell-to-cell transmission of HCV. Because host-targeting antivirals are generating enthusiastic interest for the development of treatments for hard-to-treat hepatitis C infections [81], our bioinformatics strategy is a timely approach to identify new targets for antiviral research, not only for HCV but also for other viruses as the concept of SLiM involvement in molecular mimicry is a general one.

## Methods

The four main components of our *in silico* approach are shown in Fig 1.

### A) Identify viral SLiMs

The HCV E1/E2 sequences were scanned against sequences in the ELM database (http://elm.eu.org/) [11] to find short, matching linear sequences in mammalian proteins known to interact with other mammalian proteins, which might, therefore, be mimicked by HCV E1/E2 sequences (Fig 1A). E1 and E2 sequences (from 41 and 35 HCV strains, respectively) were extracted from the UniProtKB/SwissProt database (http://www.uniprot.org/) [82] for use in our study. We required that the sequences of the matched viral SLiM needed to be conserved at a rate of at least 70%, a cutoff used for a study of HIV SLiMs by others [71] and also yielded the best performance to balance sensitivity and specificity in predicting known HCV entry factors (S5 Fig). The search retrieved 26 distinct and conserved SLiMs (Fig 1A), which were then used to find the human proteins (VIPs_direct_) that they might interact with (Fig 1B).

As shown in S10 Fig, the number of SLiMs that can be found in HCV protein sequences is roughly proportional to protein size, and all these proteins can confer the “molecular mimicry” mechanism hypothesized and be targets of our investigation; however, as explained in Introduction, we focused on E1/E2 sequences because we were primarily concerned with the viral entry process, in addition to other considerations.

### B) Construct of a virus-host PPI network

Accompanying each SLiM in the ELM database is an annotation of the protein domain(s) to which the SLiM can bind. Of the 26 identified SLiMs, 23 have information for human proteins, and of the 23, 19 were mapped to hepatocyte surface proteins that are present in the Human Integrated Protein-Protein Interaction Reference database [14] (HIPPIE release v1.7; http://cbdm-01.zdv.uni-mainz.de/~mschaefer/hippie/), which is a constantly updated human PPI database that integrate multiple experimental PPI datasets, to derive a PPI network. The list of hepatocyte surface proteins used to develop our virus-host PPI network was collected from Human Protein Reference Database [46] (HPRD; http://www.hprd.org) and Human Proteinpedia Database (http://www.humanproteinpedia.org) [47] by using the keywords “Liver” or “Hepatocyte” for the tissue type and “Extracellular region,” “Plasma membrane,” “Cell surface,” or “Cell junction” for the type of cellular component to search for potential host proteins expressed on the hepatocyte surface that might facilitate the entry of HCV. A set of 2,456 human proteins matched these keywords, and we termed this set “liver cell surface proteins”. Of these proteins, 115 (VIPs_direct_) contained at least one protein domain to which one of the 19 viral SLiMs could bind which would mimic the corresponding human SLiM. A set of 784 proteins (denoted VIPs_indirect_) were identified as binding partners to the VIPs_direct_. The interactions between the VIPs_direct_ and VIPs_indirect_, and those between the viral SLiMs and VIPs_direct_ were combined to form the virus-host network, which was then subjected to a network module/functional analysis, as described in the next step and Fig 1C.

### C) Identify network modules and their functional roles

**Fig 6:**
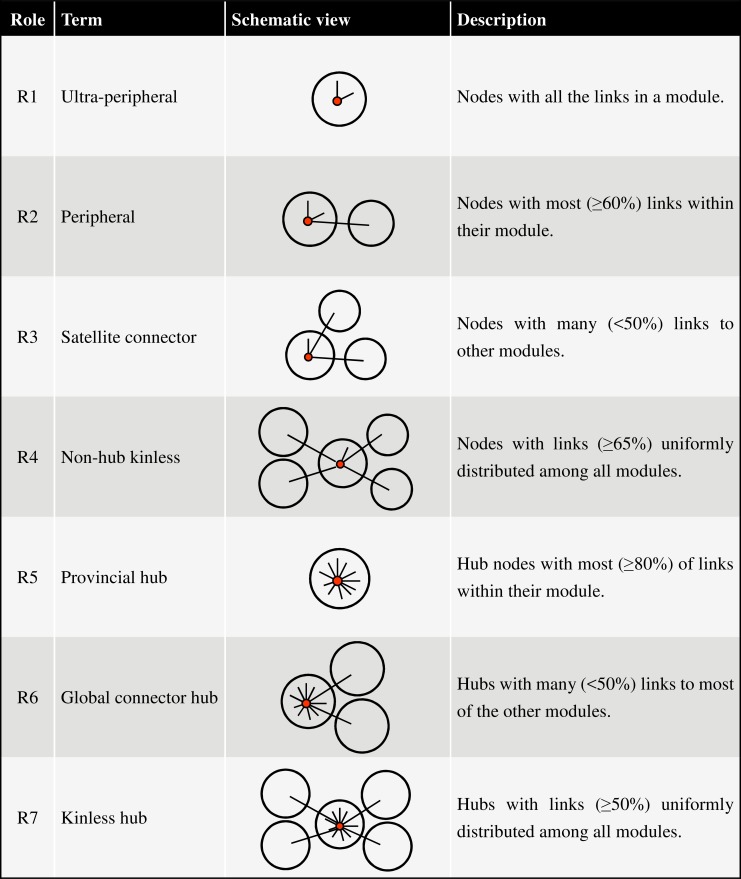
Definitions and schematic depictions of network roles. According to NetCarto [18], nodes (small red circles) with z-score of within-module degree ≥ 2.5 are defined as module hubs (nodes with many links, i.e. spikes in the schematic view), and those with z-score < 2.5 are non-hubs. Large circles represent modules. See [18] for further details.

Given a network, it is useful to determine whether it is formed of modules (i.e., communities), which are often indicative of distinct functions. For this task, we applied the network analysis tool NetCarto [18] using its large-network default settings to identify modules. The roles of importance for each network node, including every VIP, could also be assigned, which ranged from the least significant, R1 (ultra-peripheral) and R2 (peripheral), to the most significant, R6 (global connector hub) and R7 (kinless hub) (a hub is a node connected to many nodes). Following NetCarto [18], the definitions of the seven classes of network nodes and their roles in the network are summarized and schematically depicted in Fig 6.

The biological function of each network module was inferred using DAVID (https://david.ncifcrf.gov/) [83] to search for enriched GO functions [20] under the category of “biological process.” For each module, Revigo [21] was used to obtain representatives for enriched GO terms (Benjamini–Hochberg-adjusted P < 0.05), and a main functionality was deduced to cover these representatives.

### D) Build the AVP/SLiM-protein complex map

Because host VIPs_direct_ and VIPs_indirect_ may be subunits of the same protein complex targeted by the virus, we mapped the VIPs to the human protein complexes in HPRD [46] and in the mammalian protein complexes database (CORUM) [55]. The 190 complexes containing three or more VIPs were then clustered by GAP [84], a tool for matrix visualization and clustering, based on similarity related to the number of common subunits. The choice of three VIPs was made to reduce the total number of VIPs for enrichment analysis while maintaining their coverage of known HCV entry factors as much as possible. This procedure yielded six major protein-complex clusters, or groups (small groups containing fewer than five complexes were excluded), which altogether contained 177 complexes and 231 VIPs that were linked to nine viral SLiMs. The VIPs of each of the six complex groups were then subjected to KEGG pathway enrichment analysis [85] using the clusterProfiler R package [86]. A total of 43 significantly enriched hepatocyte-expressed pathways were identified (Benjamini-Hochberg-adjusted P < 0.001). In addition, a search of AVPdb [56] identified 73 peptides annotated with “Hepatitis C virus” and “Virus entry” whose inhibiting activities against HCV entry have been examined by experimental screening. Of the 73 sequences, 29 matched at least one of the nine viral SLiMs. Matching of the AVP sequences and viral SLiMs was performed at the ELM database website.

## Supporting Information

S1 Fig Statistical significance of VIPs_direct_ being hub proteins (R6 or R7) in the host PPI network. The P-value was computed using a binomial proportion test on the difference between the group of 22 R6 VIPs_direct_ and that of the remaining 93 VIPs_direct_. On top of the bar is the number of VIPs_direct_ that are also hub proteins (R6 or R7) in the host PPI network of liver cell surface proteins.
(PDF)

S2 Fig Statistical significance of overlaps between protein/gene sets. **(A)** The overlap between all VIPs and proteins in PHISTO. **(B)** The overlap between all VIPs and proteins in EHCO. **(C)** The overlap between the VIPs in the six main groups of HCV-targeted complexes and the R6 proteins. **(D)** The overlap between VIPs in the six main groups of HCV-targeted complexes and proteins in PHISTO. **(E)** The overlap between VIPs in the six main groups of HCV-targeted complexes and proteins in EHCO. **(F)** The overlap between VIPs in the six main groups of HCV-targeted complexes and known HCV entry factors. The background for panels A, B, D, and E is for all hepatocyte surface proteins and the background for panels C and F is for all VIPs.
(PDF)

S3 Fig Statistical significance of overlaps between sets of PPIs (network edges). **(A)** The overlap between HCV-VIPs_direct_ PPIs and PPIs in PHISTO that were determined as direct based on their experimental methods (see S4 Table). **(B)** The overlap between HCV-VIPs_indirect_ PPIs and PPIs in PHISTO that could not be determined as direct. Note that in PHISTO, other than core, NS3-4A and NS5A, the identity of the individual HCV protein(s) involving in the interaction with host proteins is not known; consequently, HCV was considered as a single node in the PPI network from PHISTO, and, therefore, the number of virus-host PPIs (i.e. network edges) is the same as that of the host proteins in this enrichment test.
(PDF)

S4 Fig Four known entry factors (white nodes) of HCV infection not identified in this work. **(A)** CLDN1; **(B)** SCARB1; **(C)** CD209; **(D)** NPC1L1. Blue nodes are VIPs_indirect_; gray nodes are not VIPs. The connections between the nodes represent physical interactions extracted from HIPPIE [14].
(PDF)

S5 Fig The ROC performance against known HCV entry factors of the *in silico* predictions and Li et al.’s siRNA experiment. As described in the main text, using a cutoff of at least 70% sequence conservation to find SLiMs (Fig 1A), we identified 15 of the 19 known HCV entry factors before complex analysis and 9 after, and Li et al. [5] identified 8 (see Table 1). In all, the *in silico* method identified 899 (231 after complex analysis), and Li et al.’s experiment 45, hepatocyte surface proteins as potential HCV entry factors. In this figure, the cutoff of HCV E1/E2 sequence conservation was varied from 0% to 100%, at which the same procedure as described in Fig 1 was carried out, and sensitivity and specificity for the resulting predictions were calculated to generate the ROC curves, on which the performance obtained at 70% sequence conservation cutoff is indicated. AUC: Area Under Curve.
(PDF)

S6 Fig Grouping of HCV-targeted protein complexes. Using GAP [84], 190 HCV-targeted protein complexes (containing 258 VIPs) were hierarchically clustered based on the number of shared subunits. The threshold (Cut, bottom right) for the clustering is indicated. The six main clusters (groups) are boxed and labeled A-F. Within these six groups there are 231 VIPs. *Complex ID refers to notations in S5 Table, where detailed information of the HCV-targeted complexes is provided.
(PDF)

S7 Fig VIPs versus randomly selected proteins found in protein complexes. The distribution plot illustrates 100,000 randomly sampled sets, each consisting of 899 proteins (the same number as VIPs) sampled from the set of 2,456 human hepatocyte surface proteins (Fig 1B). Each set was searched for protein complexes, which were required to contain at least three sampled proteins as indicated in Fig 1D. The number of proteins retained in their targeted complexes in each sampling was counted as “number of sampled proteins in complexes”. The observed number (i.e., 258, indicated by dotted line) is the number of the VIPs found in the 190 HCV-targeted protein complexes.
(PDF)

S8 Fig Statistical significance of the three main enriched functionalities of HCV E1/E2-derived VIPs compared to those containing a binding domain for any SLiM in the ELM database. The distribution plots for the functionality of **(A)** *entry*, **(B)** *carcinogenesis*, and **(C)** *infectious disease* were derived from results of 10,000 randomly sampled sets of proteins. In each set, 899 proteins (the same number as VIPs) were randomly sampled from a set of 1,320 proteins, which is the number of proteins containing a binding domain for any SLiM in the ELM database (348 proteins) and their first PPI neighbors (972 proteins) in the human PPI network of liver cell surface proteins. The same procedure as described in Fig 1D (see Methods) for protein complex and KEGG pathway analyses was carried out for each sample set. The number of enriched KEGG pathways in functionality of *entry*, *carcinogenesis*, and *infectious disease*, was counted respectively. The observed number (indicated by dotted line) is the number of KEGG pathways belonging to the given functionality enriched in the set of VIPs found in the six main groups of HCV-targeted protein complexes (Fig 4).
(PDF)

S9 Fig Statistical significance of SLiM-containing AVPs. **(A)** Of the 73 AVPs that were tested for entry-related anti-HCV activities in AVPdb, 29 harbor an HCV E1/E2 SLiM (one of nine) that can bind to VIPs found in the six main groups of HCV-targeted protein complexes (Fig 4); here, the number of AVPs containing at least one of the nine SLiMs for all sample sets, each consisting of 73 AVPs randomly sampled from a pool of 2059 AVPs (the total number of AVPs in AVPdb), was counted. **(B)** In this test, the sampling pool was the set of the 431 AVPs found to contain LIG_SH2_STAT5 and/or LIG_SH3_3 in AVPdb; the sample size was 23, of which those shown to be effective in inhibiting HCV entry in AVPdb were counted. **(C)** In this test, the sampling pool was the set of the 154 AVPs found to contain MOD_ProDKin_1 in AVPdb; the sample size was 7, of which those shown to be effective in inhibiting HCV entry in AVPdb were counted. For (A), (B) and (C), the statistics was calculated based on 10,000 sample sets.
(PDF)

S10 Fig The number of conserved (in ≥ 70% sequences) SLiMs found in HCV component proteins and protein length. (PDF)

S1 Table GO terms enriched in the virus-host PPI network modules and representative functions according to Revigo. (XLSX)

S2 Table Representative function(s) and supporting published experimental data for HCV-human PPI network modules 1–7. (PDF)

S3 Table Published experimental evidence for relations between SLiMs and R6 proteins and between R6 proteins and module functions in Fig 3. (PDF)

S4 Table Types of PPIs between HCV polyprotein and human protein in PHISTO and their detection methods. (PDF)

S5 Table HCV-targeted protein complexes. (XLSX)

S6 Table KEGG pathways enriched in the six main protein complex groups. (PDF)

S1 Appendix Main functionalities associated with KEGG pathways enriched in the HCV-targeted protein complexes. (PDF)
