# Supplementary material for: Identification of Entry Factors Involved in Hepatitis C Virus Infection Based on Host-Mimicking Short Linear Motifs
Source: PLoS Comput Biol. 2017 Jan 27;13(1):e1005368. doi: 10.1371/journal.pcbi.1005368 (PMC5302801; doi:10.1371/journal.pcbi.1005368)
Supplement: S1 Fig — The P-value was computed using a binomial proportion test on the difference between the group of 22 R6 VIPsdirect and that of the remaining 93 VIPsdirect. On top of the bar is the number of VIPsdirect that are also hub proteins (R6 or R7) in the host PPI network of liver cell surface proteins. (PDF) [file pcbi.1005368.s001.pdf]

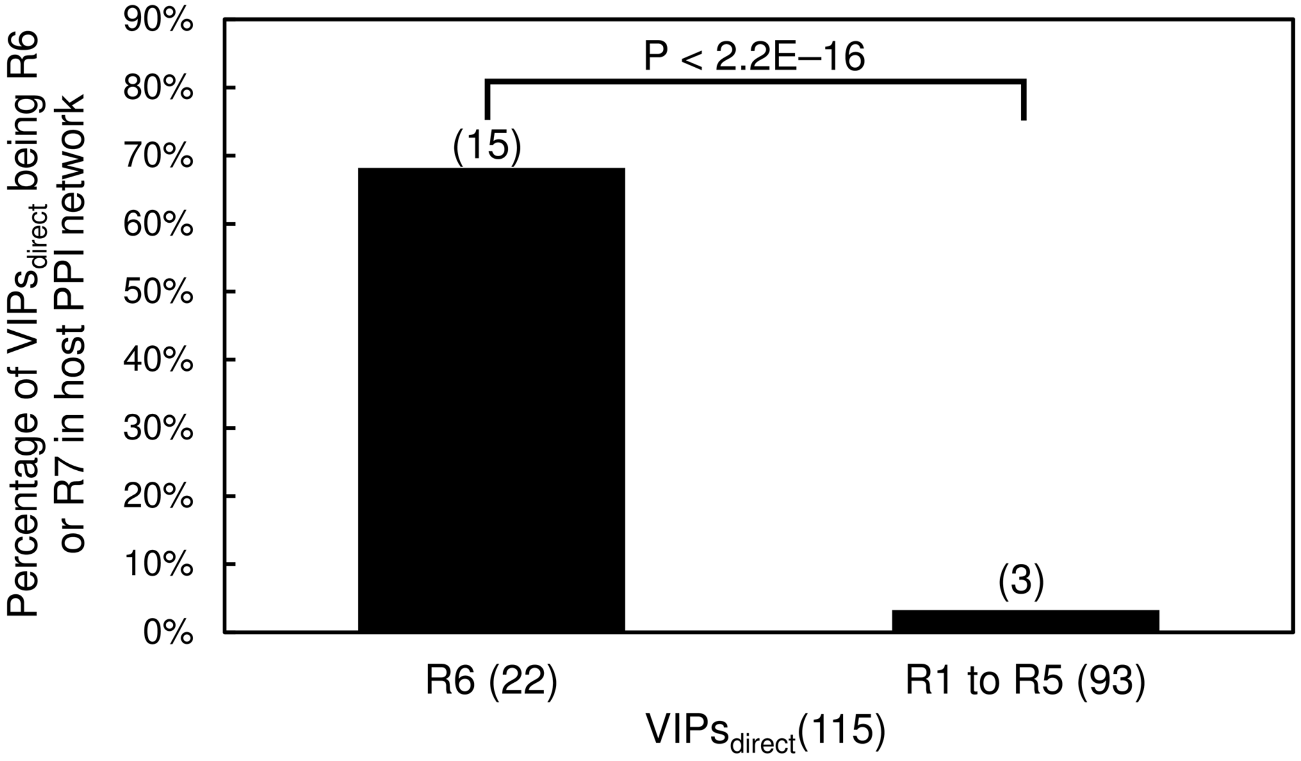

S1 Fig. Statistical significance of VIPs$_{direct}$ being hub proteins (R6 or R7) in the host PPI network. The P-value was computed using a binomial proportion test on the difference between the group of 22 R6 VIPs$_{direct}$ and that of the remaining 93 VIPs$_{direct}$. On top of the bar is the number of VIPs$_{direct}$ that are also hub proteins (R6 or R7) in the host PPI network of liver cell surface proteins.
