# Supplementary material for: Identification of Entry Factors Involved in Hepatitis C Virus Infection Based on Host-Mimicking Short Linear Motifs
Source: PLoS Comput Biol. 2017 Jan 27;13(1):e1005368. doi: 10.1371/journal.pcbi.1005368 (PMC5302801; doi:10.1371/journal.pcbi.1005368)
Supplement: S2 Fig — (A) The overlap between all VIPs and proteins in PHISTO. (B) The overlap between all VIPs and proteins in EHCO. (C) The overlap between the VIPs in the six main groups of HCV-targeted complexes and the R6 proteins. (D) The overlap between VIPs in the six main groups of HCV-targeted complexes and proteins in PHISTO. (E) The overlap between VIPs in the six main groups of HCV-targeted complexes and proteins in EHCO. (F) The overlap between VIPs in the six main groups of HCV-targeted complexes and known HCV entry factors. The background for panels A, B, D, and E is for all hepatocyte surface proteins and the background for panels C and F is for all VIPs. (PDF) [file pcbi.1005368.s002.pdf]

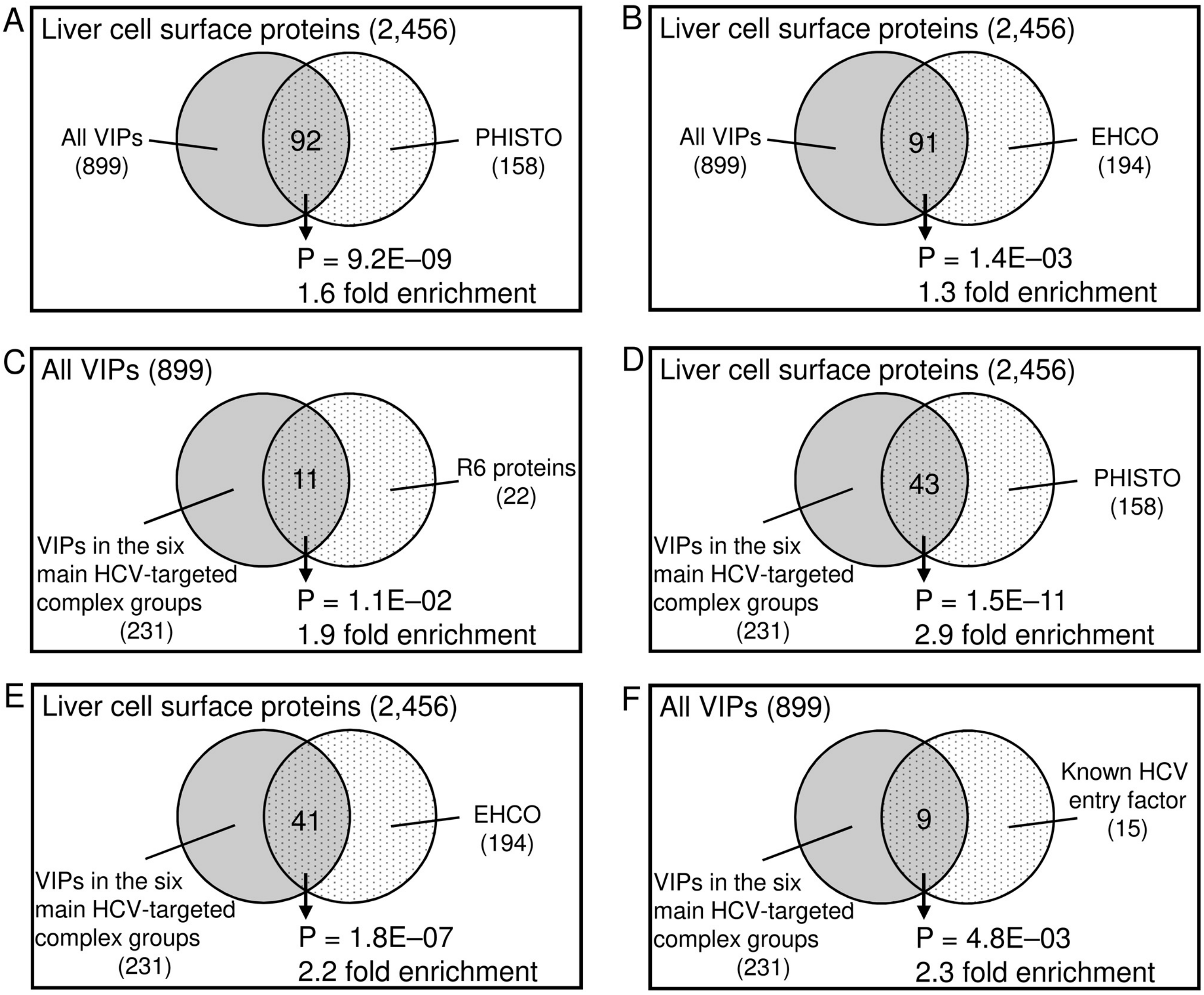

S2 Fig. Statistical significance of overlaps between protein/gene sets. (A) The overlap between all VIPs and proteins in PHISTO. **(B)** The overlap between all VIPs and proteins in EHCO. **(C)** The overlap between the VIPs in the six main groups of HCV-targeted complexes and the R6 proteins. **(D)** The overlap between VIPs in the six main groups of HCV-targeted complexes and proteins in PHISTO. **(E)** The overlap between VIPs in the six main groups of HCV-targeted complexes and proteins in EHCO. **(F)** The overlap between VIPs in the six main groups of HCV-targeted complexes and known HCV entry factors. The background for panels A, B, D, and E is for all hepatocyte surface proteins and the background for panels C and F is for all VIPs.
