# Supplementary material for: Identification of Entry Factors Involved in Hepatitis C Virus Infection Based on Host-Mimicking Short Linear Motifs
Source: PLoS Comput Biol. 2017 Jan 27;13(1):e1005368. doi: 10.1371/journal.pcbi.1005368 (PMC5302801; doi:10.1371/journal.pcbi.1005368)
Supplement: S3 Fig — (A) The overlap between HCV-VIPsdirect PPIs and PPIs in PHISTO that were determined as direct based on their experimental methods (see S4 Table). (B) The overlap between HCV-VIPsindirect PPIs and PPIs in PHISTO that could not be determined as direct. Note that in PHISTO, other than core, NS3-4A and NS5A, the identity of the individual HCV protein(s) involving in the interaction with host proteins is not known; consequently, HCV was considered as a single node in the PPI network from PHISTO, and, therefore, the number of virus-host PPIs (i.e. network edges) is the same as that of the host proteins in this enrichment test. (PDF) [file pcbi.1005368.s003.pdf]

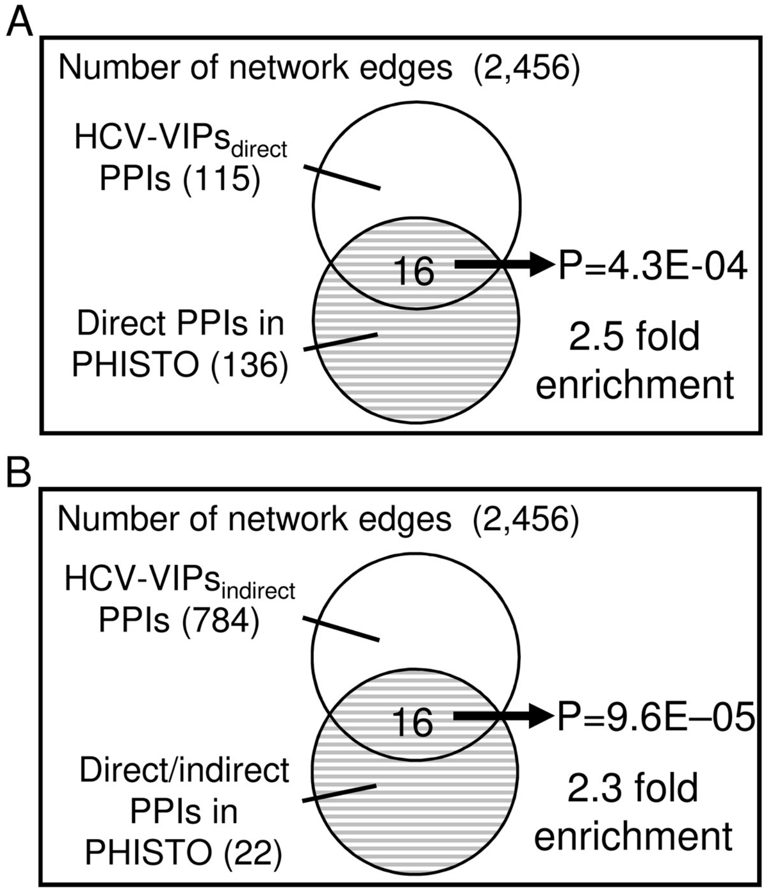

S3 Fig. Statistical significance of overlaps between sets of PPIs (network edges). (A) The overlap between HCV-VIPs$_{direct}$ PPIs and PPIs in PHISTO that were determined as direct based on their experimental methods (see S4 Table). **(B)** The overlap between HCV-VIPs$_{indirect}$ PPIs and PPIs in PHISTO that could not be determined as direct. Note that in PHISTO, other than core, NS3-4A and NS5A, the identity of the individual HCV protein(s) involving in the interaction with host proteins is not known; consequently, HCV was considered as a single node in the PPI network from PHISTO, and, therefore, the number of virus-host PPIs (i.e. network edges) is the same as that of the host proteins in this enrichment test.
