# Supplementary material for: Identification of Entry Factors Involved in Hepatitis C Virus Infection Based on Host-Mimicking Short Linear Motifs
Source: PLoS Comput Biol. 2017 Jan 27;13(1):e1005368. doi: 10.1371/journal.pcbi.1005368 (PMC5302801; doi:10.1371/journal.pcbi.1005368)
Supplement: S4 Fig — (A) CLDN1; (B) SCARB1; (C) CD209; (D) NPC1L1. Blue nodes are VIPsindirect; gray nodes are not VIPs. The connections between the nodes represent physical interactions extracted from HIPPIE [14]. (PDF) [file pcbi.1005368.s004.pdf]

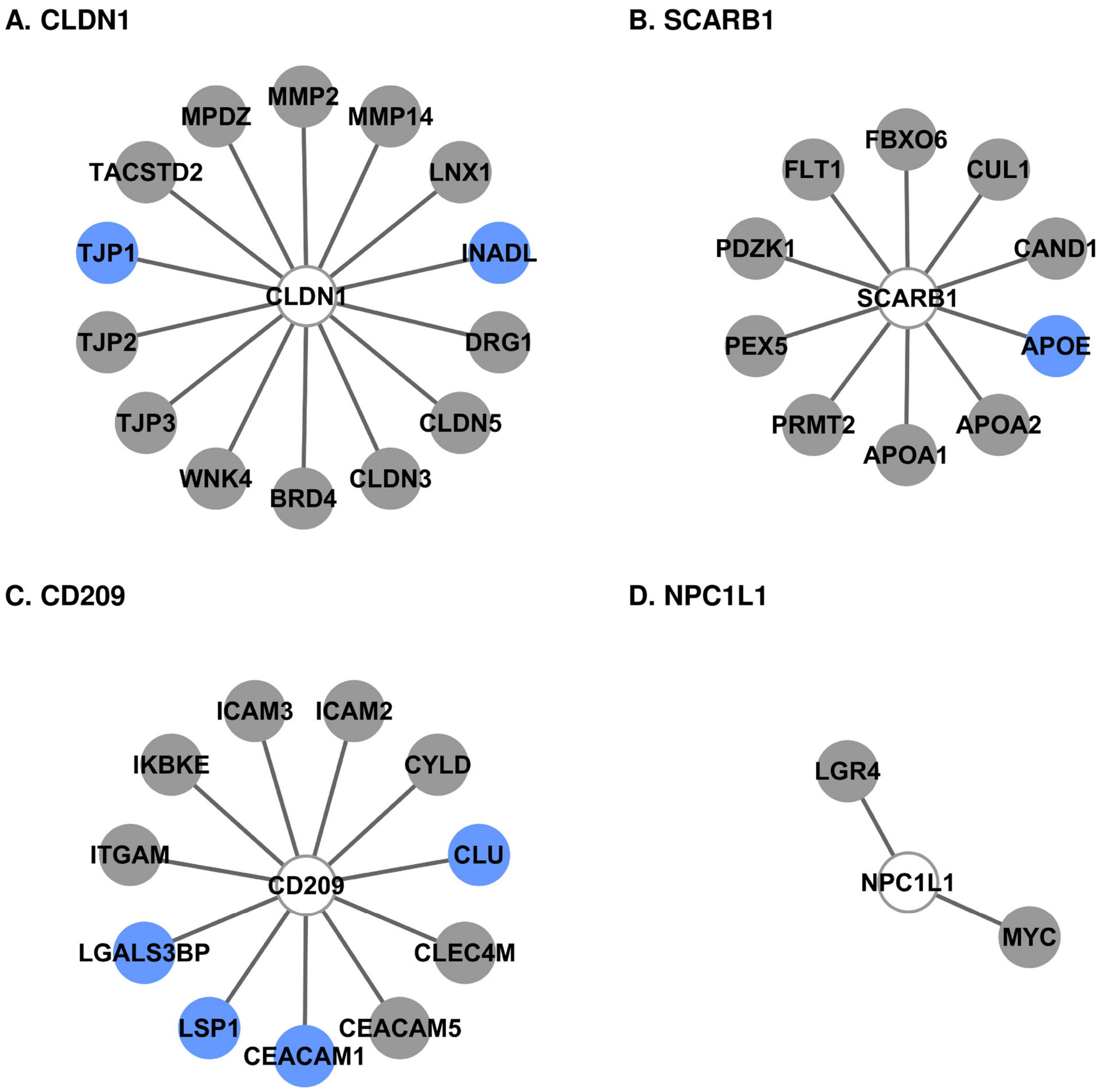

S4 Fig. Four known entry factors (white nodes) of HCV infection not identified in this work. (A) CLDN1; **(B)** SCARB1; **(C)** CD209; **(D)** NPC1L1. Blue nodes are VIPs$_{\text{indirect}}$; gray nodes are not VIPs. The connections between the nodes represent physical interactions extracted from HIPPIE (Schaefer et al. (2012) *PLoS ONE*, *7(2)*, e31826).
