# Supplementary material for: Identification of Entry Factors Involved in Hepatitis C Virus Infection Based on Host-Mimicking Short Linear Motifs
Source: PLoS Comput Biol. 2017 Jan 27;13(1):e1005368. doi: 10.1371/journal.pcbi.1005368 (PMC5302801; doi:10.1371/journal.pcbi.1005368)
Supplement: S5 Fig — As described in the main text, using a cutoff of at least 70% sequence conservation to find SLiMs (Fig 1A), we identified 15 of the 19 known HCV entry factors before complex analysis and 9 after, and Li et al. [5] identified 8 (see Table 1). In all, the in silico method identified 899 (231 after complex analysis), and Li et al.’s experiment 45, hepatocyte surface proteins as potential HCV entry factors. In this figure, the cutoff of HCV E1/E2 sequence conservation was varied from 0% to 100%, at which the same procedure as described in Fig 1 was carried out, and sensitivity and specificity for the resulting predictions were calculated to generate the ROC curves, on which the performance obtained at 70% sequence conservation cutoff is indicated. AUC: Area Under Curve. (PDF) [file pcbi.1005368.s005.pdf]

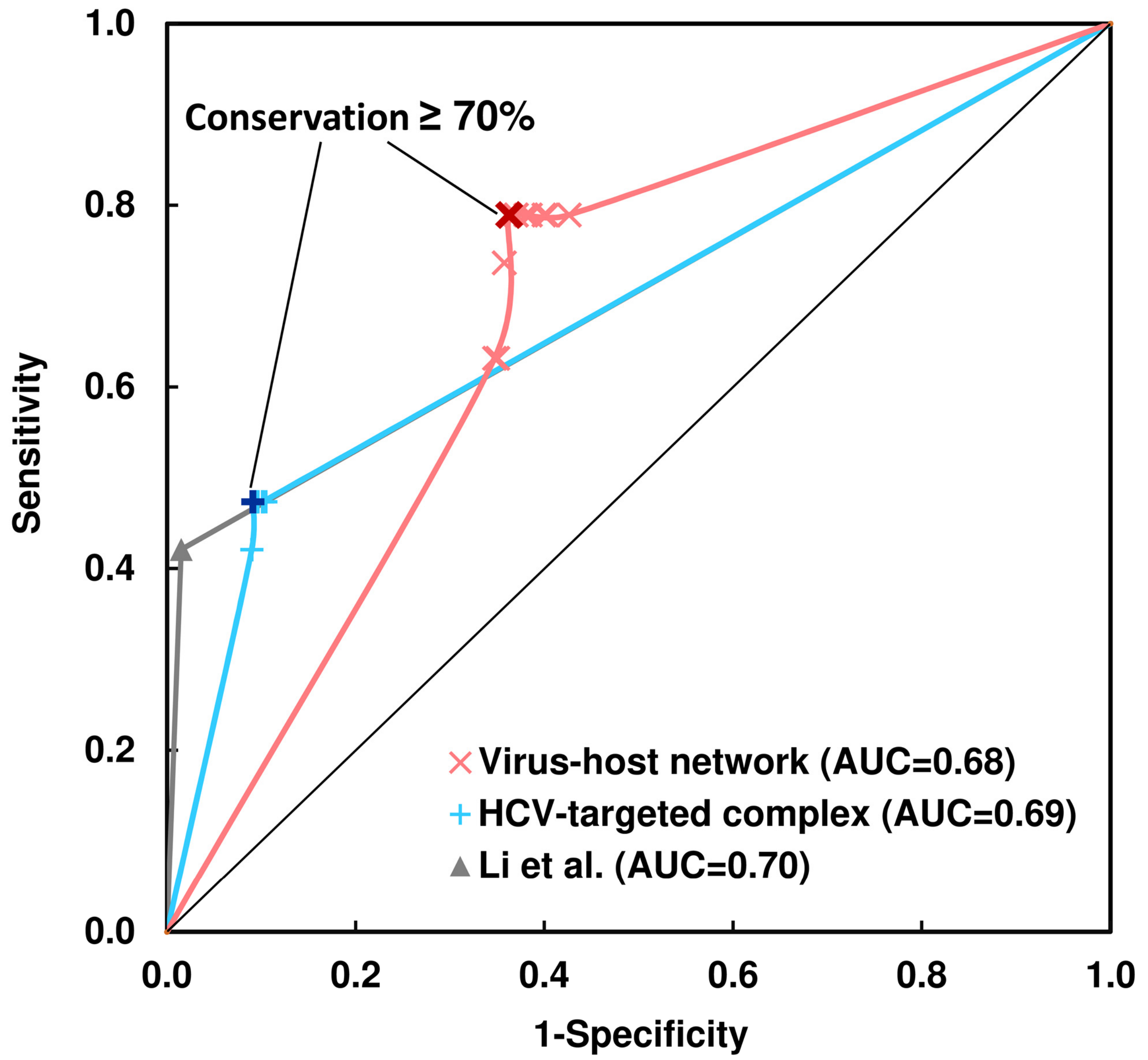

S5 Fig. The ROC performance against known HCV entry factors of the *in silico* predictions and Li et al.'s siRNA experiment. As described in the main text, using a cutoff of at least 70% sequence conservation to find SLiMs (Fig 1A), we identified 15 of the 19 known HCV entry factors before complex analysis and 9 after, and Li et al. (Li et al. (2014) *PLoS Pathog*, *10(5)*, e1004163) identified 8 (see Table 1). In all, the *in silico* method identified 899 (231 after complex analysis), and Li et al.'s experiment 45, hepatocyte surface proteins as potential HCV entry factors. In this figure, the cutoff of HCV E1/E2 sequence conservation was varied from 0% to 100%, at which the same procedure as described in Fig 1 was carried out, and sensitivity and specificity for the resulting predictions were calculated to generate the ROC curves, on which the performance obtained at 70% sequence conservation cutoff is indicated. AUC: Area Under Curve.
