# Supplementary material for: Identification of Entry Factors Involved in Hepatitis C Virus Infection Based on Host-Mimicking Short Linear Motifs
Source: PLoS Comput Biol. 2017 Jan 27;13(1):e1005368. doi: 10.1371/journal.pcbi.1005368 (PMC5302801; doi:10.1371/journal.pcbi.1005368)
Supplement: S6 Fig — Using GAP [84], 190 HCV-targeted protein complexes (containing 258 VIPs) were hierarchically clustered based on the number of shared subunits. The threshold (Cut, bottom right) for the clustering is indicated. The six main clusters (groups) are boxed and labeled A-F. Within these six groups there are 231 VIPs. *Complex ID refers to notations in S5 Table, where detailed information of the HCV-targeted complexes is provided. (PDF) [file pcbi.1005368.s006.pdf]

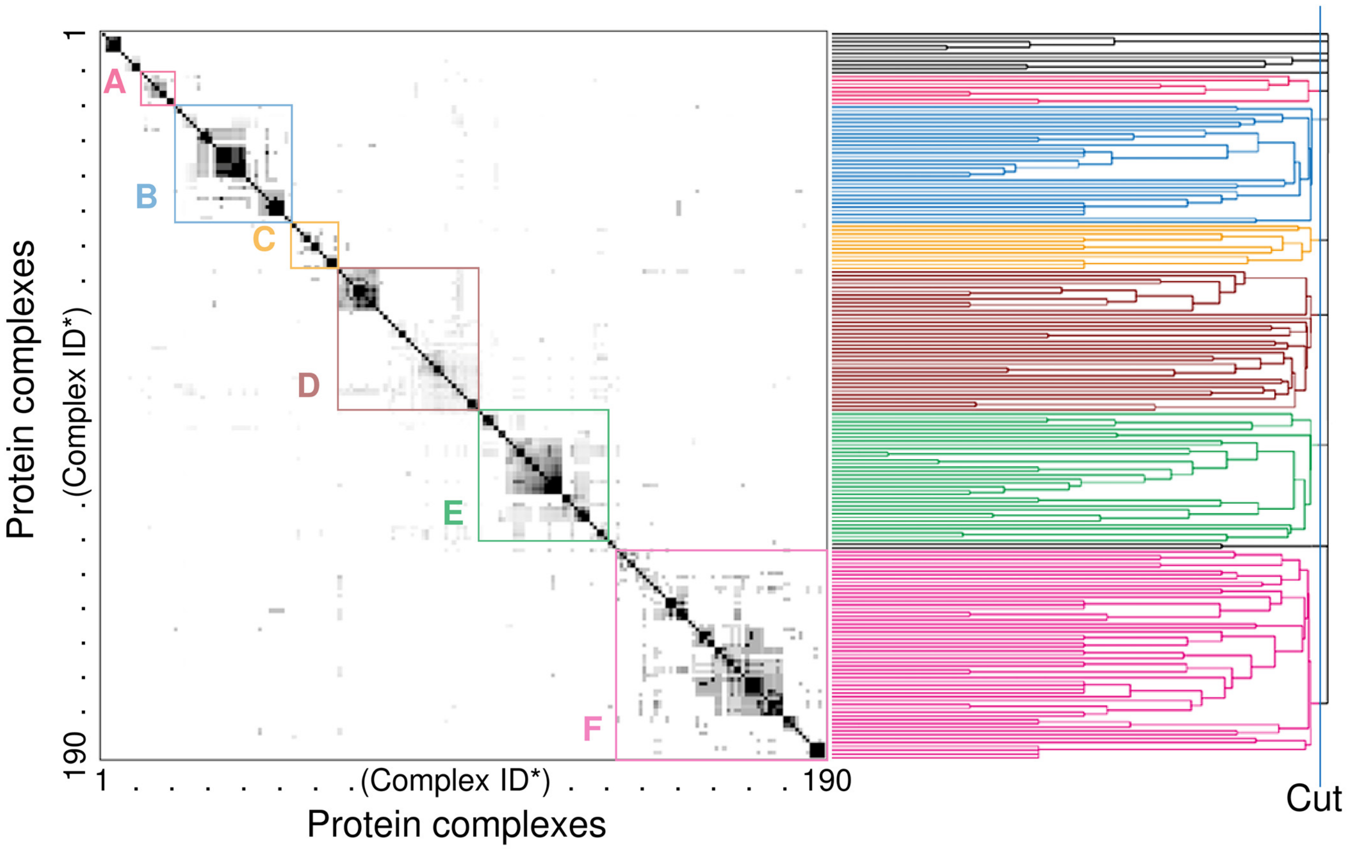

S6 Fig. Grouping of HCV-targeted protein complexes. Using GAP (Wu et al. (2010) *Computational Statistics & Data Analysis*, *54*(3), 767–778), 190 HCV-targeted protein complexes (containing 258 VIPs) were hierarchically clustered based on the number of shared subunits. The threshold (Cut, bottom right) for the clustering is indicated. The six main clusters (groups) are boxed and labeled A-F. Within these six groups there are 231 VIPs. *Complex ID refers to notations in S5 Table, where detailed information of the HCV-targeted complexes is provided.
