# Supplementary material for: Identification of Entry Factors Involved in Hepatitis C Virus Infection Based on Host-Mimicking Short Linear Motifs
Source: PLoS Comput Biol. 2017 Jan 27;13(1):e1005368. doi: 10.1371/journal.pcbi.1005368 (PMC5302801; doi:10.1371/journal.pcbi.1005368)
Supplement: S7 Fig — The distribution plot illustrates 100,000 randomly sampled sets, each consisting of 899 proteins (the same number as VIPs) sampled from the set of 2,456 human hepatocyte surface proteins (Fig 1B). Each set was searched for protein complexes, which were required to contain at least three sampled proteins as indicated in Fig 1D. The number of proteins retained in their targeted complexes in each sampling was counted as “number of sampled proteins in complexes”. The observed number (i.e., 258, indicated by dotted line) is the number of the VIPs found in the 190 HCV-targeted protein complexes. (PDF) [file pcbi.1005368.s007.pdf]

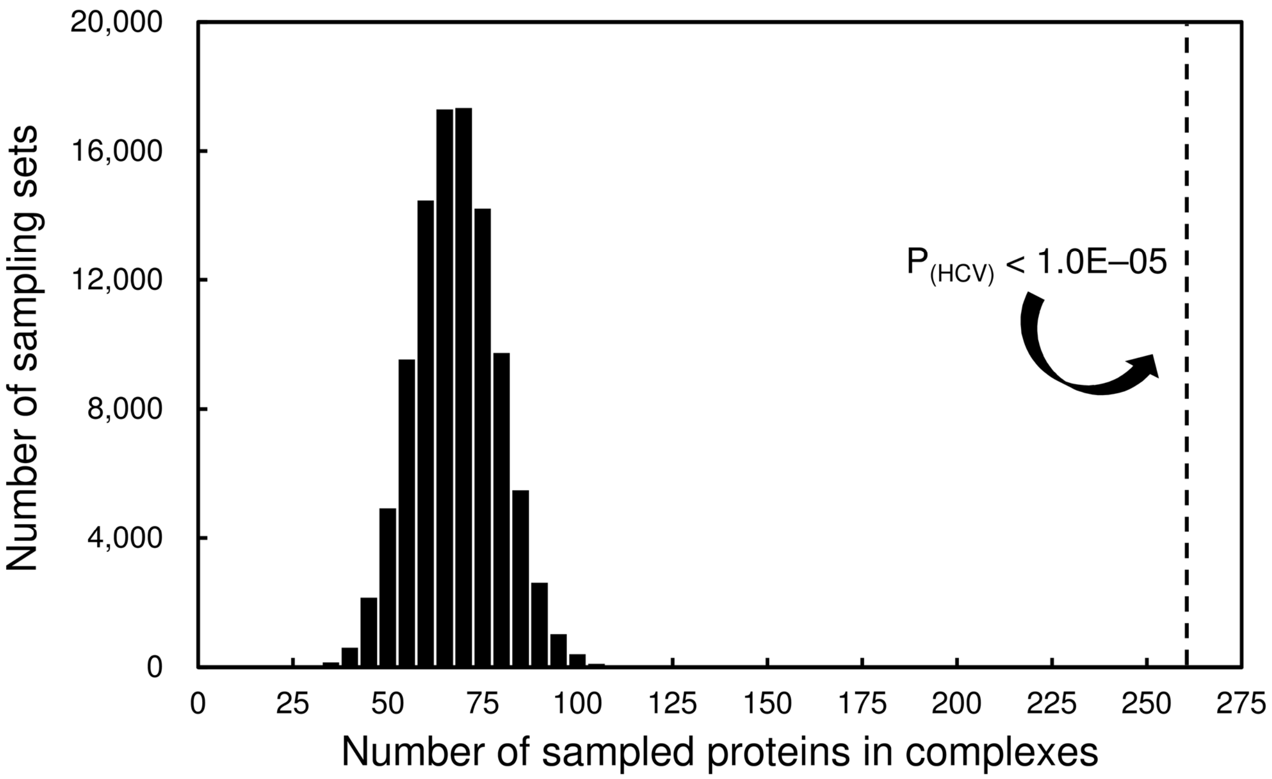

S7 Fig. VIPs versus randomly selected proteins found in protein complexes. The distribution plot illustrates 100,000 randomly sampled sets, each consisting of 899 proteins (the same number as VIPs) sampled from the set of 2,456 human hepatocyte surface proteins (Fig 1B). Each set was searched for protein complexes, which were required to contain at least three sampled proteins as indicated in Fig 1D. The number of proteins retained in their targeted complexes in each sampling was counted as "number of sampled proteins in complexes". The observed number (i.e., 258, indicated by dotted line) is the number of the VIPs found in the 190 HCV-targeted protein complexes.
