# Supplementary material for: Identification of Entry Factors Involved in Hepatitis C Virus Infection Based on Host-Mimicking Short Linear Motifs
Source: PLoS Comput Biol. 2017 Jan 27;13(1):e1005368. doi: 10.1371/journal.pcbi.1005368 (PMC5302801; doi:10.1371/journal.pcbi.1005368)
Supplement: S8 Fig — The distribution plots for the functionality of (A) entry, (B) carcinogenesis, and (C) infectious disease were derived from results of 10,000 randomly sampled sets of proteins. In each set, 899 proteins (the same number as VIPs) were randomly sampled from a set of 1,320 proteins, which is the number of proteins containing a binding domain for any SLiM in the ELM database (348 proteins) and their first PPI neighbors (972 proteins) in the human PPI network of liver cell surface proteins. The same procedure as described in Fig 1D (see Methods) for protein complex and KEGG pathway analyses was carried out for each sample set. The number of enriched KEGG pathways in functionality of entry, carcinogenesis, and infectious disease, was counted respectively. The observed number (indicated by dotted line) is the number of KEGG pathways belonging to the given functionality enriched in the set of VIPs found in the six main groups of HCV-targeted protein complexes (Fig 4). (PDF) [file pcbi.1005368.s008.pdf]

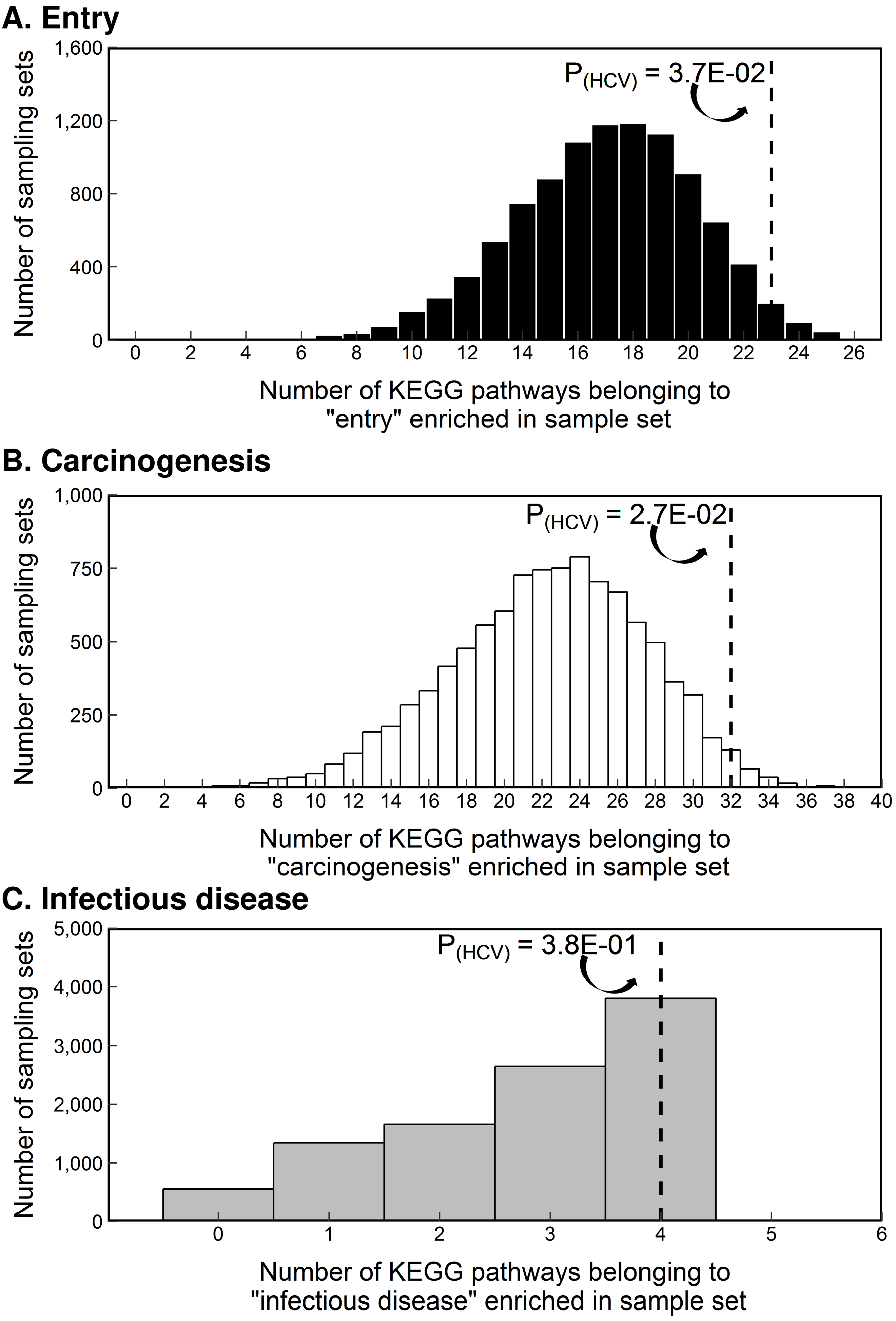
A. Entry
Number of sampling sets
1,600
1,200
800
400
0
P(HCV) = 3.7E-02
0 2 4 6 8 10 12 14 16 18 20 22 24 26
Number of KEGG pathways belonging to
"entry" enriched in sample set
B. Carcinogenesis
Number of sampling sets
1,000
750
500
250
0
P(HCV) = 2.7E-02
0 2 4 6 8 10 12 14 16 18 20 22 24 26 28 30 32 34 36 38 40
Number of KEGG pathways belonging to
"carcinogenesis" enriched in sample set
C. Infectious disease
Number of sampling sets
5,000
4,000
3,000
2,000
1,000
0
P(HCV) = 3.8E-01
0 1 2 3 4 5 6
Number of KEGG pathways belonging to
"infectious disease" enriched in sample set

S8 Fig. Statistical significance of the three main enriched functionalities of HCV E1/E2-derived VIPs compared to those containing a binding domain for any SLiM in the ELM database. The distribution plots for the functionality of **(A)** *entry*, **(B)** *carcinogenesis*, and **(C)** *infectious disease* were derived from results of 10,000 randomly sampled sets of proteins. In each set, 899 proteins (the same number as VIPs) were randomly sampled from a set of 1,320 proteins, which is the number of proteins containing a binding domain for any SLiM in the ELM database (348 proteins) and their first PPI neighbors (972 proteins) in the human PPI network of liver cell surface proteins. The same procedure as described in Fig 1D (see Methods) for protein complex and KEGG pathway analyses was carried out for each sample set. The number of enriched KEGG pathways in functionality of *entry*, *carcinogenesis*, and *infectious disease*, was counted respectively. The observed number (indicated by dotted line) is the number of KEGG pathways belonging to the given functionality enriched in the set of VIPs found in the six main groups of HCV-targeted protein complexes (Fig. 4).
