# Supplementary material for: Identification of Entry Factors Involved in Hepatitis C Virus Infection Based on Host-Mimicking Short Linear Motifs
Source: PLoS Comput Biol. 2017 Jan 27;13(1):e1005368. doi: 10.1371/journal.pcbi.1005368 (PMC5302801; doi:10.1371/journal.pcbi.1005368)
Supplement: S9 Fig — (A) Of the 73 AVPs that were tested for entry-related anti-HCV activities in AVPdb, 29 harbor an HCV E1/E2 SLiM (one of nine) that can bind to VIPs found in the six main groups of HCV-targeted protein complexes (Fig 4); here, the number of AVPs containing at least one of the nine SLiMs for all sample sets, each consisting of 73 AVPs randomly sampled from a pool of 2059 AVPs (the total number of AVPs in AVPdb), was counted. (B) In this test, the sampling pool was the set of the 431 AVPs found to contain LIG_SH2_STAT5 and/or LIG_SH3_3 in AVPdb; the sample size was 23, of which those shown to be effective in inhibiting HCV entry in AVPdb were counted. (C) In this test, the sampling pool was the set of the 154 AVPs found to contain MOD_ProDKin_1 in AVPdb; the sample size was 7, of which those shown to be effective in inhibiting HCV entry in AVPdb were counted. For (A), (B) and (C), the statistics was calculated based on 10,000 sample sets. (PDF) [file pcbi.1005368.s009.pdf]

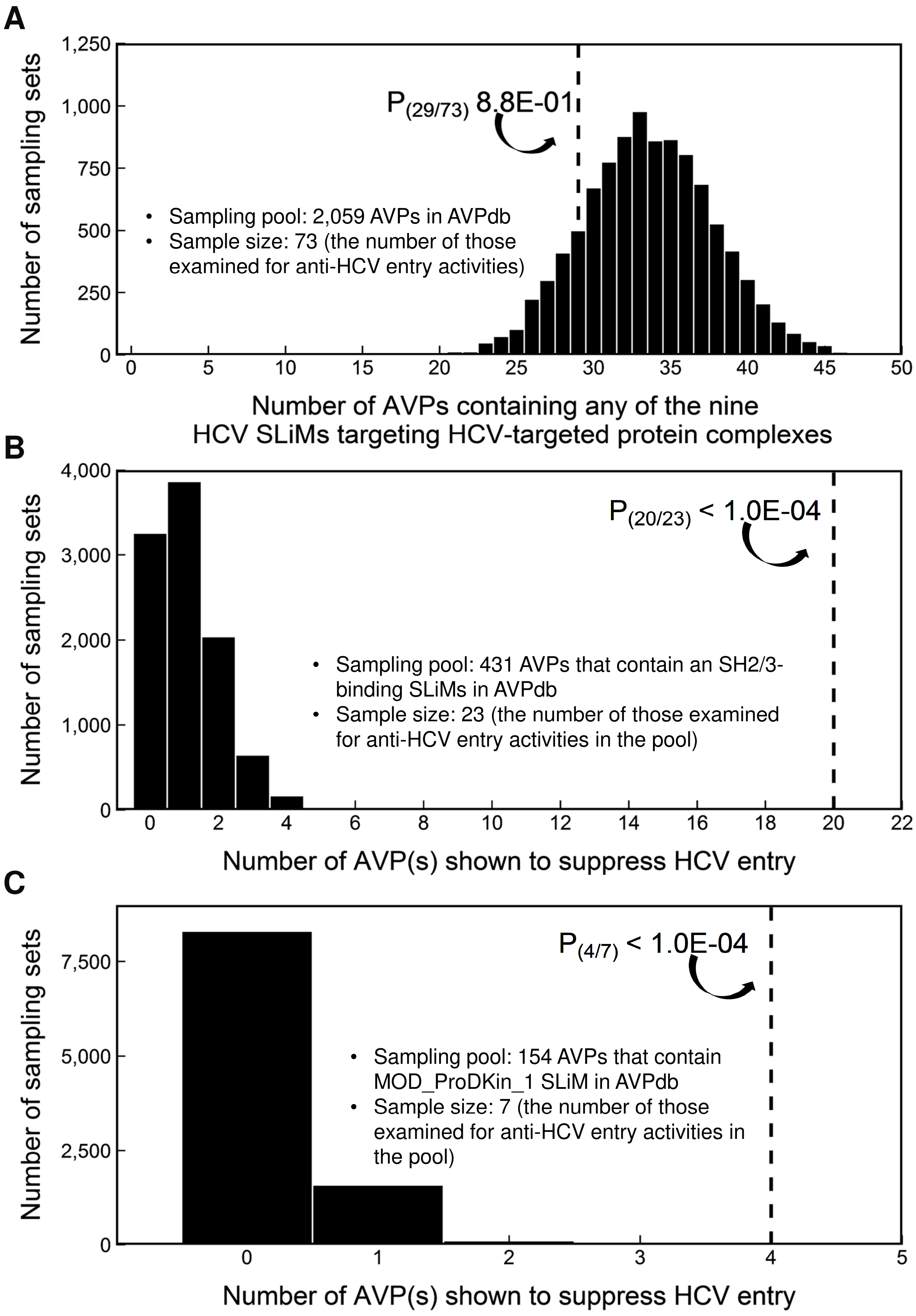

A
Number of sampling sets
P(29/73) 8.8E-01
• Sampling pool: 2,059 AVPs in AVPdb
• Sample size: 73 (the number of those examined for anti-HCV entry activities)
Number of AVPs containing any of the nine HCV SLiMs targeting HCV-targeted protein complexes
B
Number of sampling sets
P(20/23) < 1.0E-04
• Sampling pool: 431 AVPs that contain an SH2/3-binding SLiMs in AVPdb
• Sample size: 23 (the number of those examined for anti-HCV entry activities in the pool)
Number of AVP(s) shown to suppress HCV entry
C
Number of sampling sets
P(4/7) < 1.0E-04
• Sampling pool: 154 AVPs that contain MOD_ProDKin_1 SLiM in AVPdb
• Sample size: 7 (the number of those examined for anti-HCV entry activities in the pool)
Number of AVP(s) shown to suppress HCV entry

S9 Fig. Statistical significance of SLiM-containing AVPs. (A) Of the 73 AVPs that were tested for entry-related anti-HCV activities in AVPdb, 29 harbor an HCV E1/E2 SLiM (one of nine) that can bind to VIPs found in the six main groups of HCV-targeted protein complexes (Fig. 4); here, the number of AVPs containing at least one of the nine SLiMs for all sample sets, each consisting of 73 AVPs randomly sampled from a pool of 2059 AVPs (the total number of AVPs in AVPdb), was counted. **(B)** In this test, the sampling pool was the set of the 431 AVPs found to contain LIG_SH2_STAT5 and/or LIG_SH3_3 in AVPdb; the sample size was 23, of which those shown to be effective in inhibiting HCV entry in AVPdb were counted. **(C)** In this test, the sampling pool was the set of the 154 AVPs found to contain MOD_ProDKin_1 in AVPdb; the sample size was 7, of which those shown to be effective in inhibiting HCV entry in AVPdb were counted. For (A), (B) and (C), the statistics was calculated based on 10,000 sample sets.
