# Supplementary material for: Identification of Entry Factors Involved in Hepatitis C Virus Infection Based on Host-Mimicking Short Linear Motifs
Source: PLoS Comput Biol. 2017 Jan 27;13(1):e1005368. doi: 10.1371/journal.pcbi.1005368 (PMC5302801; doi:10.1371/journal.pcbi.1005368)
Supplement: S10 Fig — (PDF) [file pcbi.1005368.s010.pdf]

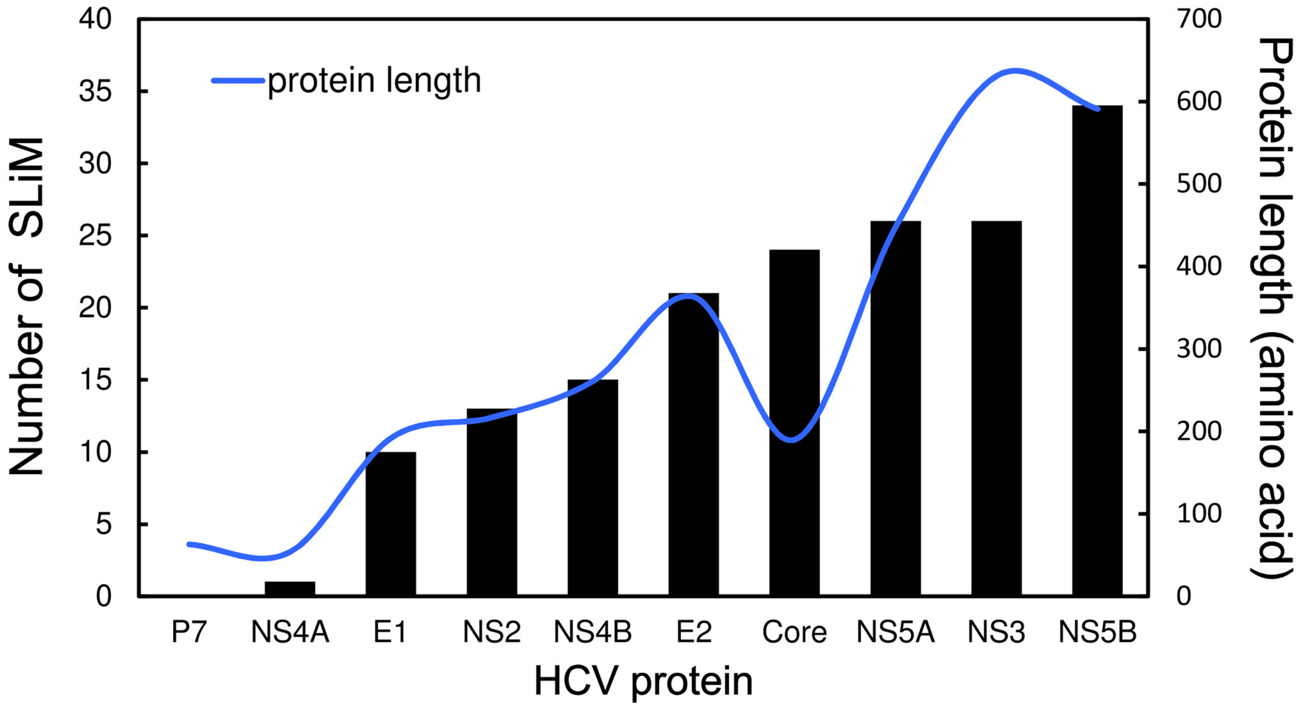

S10 Fig. The number of conserved (in ≥ 70% sequences) SLiMs found in HCV component proteins and protein length.
