# Supplementary material for: Identification of Entry Factors Involved in Hepatitis C Virus Infection Based on Host-Mimicking Short Linear Motifs
Source: PLoS Comput Biol. 2017 Jan 27;13(1):e1005368. doi: 10.1371/journal.pcbi.1005368 (PMC5302801; doi:10.1371/journal.pcbi.1005368)
Supplement: S2 Table — (PDF) [file pcbi.1005368.s012.pdf]

S2 Table. Representative function(s) and supporting published experimental data for HCV-human PPI network modules 1–7

**Network module**	**Description (literature evidence)**
Module 1: Actin cytoskeleton organization and cell adhesion	(a) HCV E1/E2 can promote actin reorganization to induce internalization of the virion[1].<br>(b) Adhesion between host cells can prevent invasion of pathogens[2]. However, during HCV entry, OCLN, a cell adhesion protein, interacts with HCV E2[3] and relocalizes HCV to tight junctions where HCV internalization occurs[1, 4, 5].
Module 2: Peptide hormone processing	HCV infection has been associated with insulin resistance, a risk factor for hepatocellular carcinoma (HCC)[6].
Module 3: Cellular homeostasis	HCV E1/E2 can trigger the unfolded protein response (UPR)[7], a cellular homeostatic process triggered in response to stress induced by unfolded/misfolded proteins at the endoplasmic reticulum[8]. HCV-triggered UPR can lead to autophagy, which is also a cellular homeostatic response[9].
Module 4: Growth	HCV infection enhances the growth of HCC[10]. For instance, in HCV-associated cirrhosis tissues, upregulation of IGFBP3, an IGF-binding protein, can potentiate IGF signaling and contribute to tumor growth[11, 12].
Module 5: Apoptosis and cell junction organization	(a) During HCV infection, apoptosis in infected cells is triggered to decrease the spread of the virus[13, 14]. However, HCV has evolved several anti-apoptotic strategies to increase its survival: 1) HCV E1 induces the production of reactive oxygen species (ROS) and the phosphorylation of STAT3[15], leading to cell survival; 2) HCV infection activates CHUK[16], which can upregulate the activity of NF-κB and lead to enhancement of the expression of anti-apoptotic genes[17]; 3) HCV E1/E2 can induce the phosphorylation of AKT proteins and activate PIK3-AKT signaling to enhance cell survival[18].<br>(b) Based on an *in vitro* study, HCV infection can regulate tight junction organization by downregulating the expression of CLDN1 and OCLN, which may lead to the observed morphological and functional alterations of HCV-infected hepatocytes[19].
Module 6: Endocytosis and cell-cell signaling	(a) Several studies have demonstrated that clathrin-dependent endocytosis is the main route for HCV to enter human hepatocytes, mediated by HCV E1/E2[20-22].<br>(b) Upon HCV infection, cell-cell signaling in the host cell can be triggered to induce a systematic immune response against viral infection[23].
Module 7: Receptor signaling and cytoskeleton organization	(a) Receptor signaling, e.g., EGFR signaling and its downstream signaling by HRAS[24] and PI3K-AKT[18] are pivotal to HCV entry[24]. These signaling events stimulate hepatocyte proliferation, which may contribute to hepatocellular carcinogenesis[25, 26].<br>(c) During HCV entry, the virus induces cytoskeleton reorganization so that it is relocalized to the tight junction, where internalization and endocytosis occur[1, 4, 5]. PTPN11 upregulated by HCV infection [27] may reorganize the cytoskeleton[28].

S2 Table references

1. Brazzoli M, Bianchi A, Filippini S, Weiner A, Zhu Q, Pizza M, et al. CD81 is a central regulator of cellular events required for hepatitis C virus infection of human hepatocytes. J Virol. 2008;82(17):8316-29.
2. Bonazzi M, Cossart P. Impenetrable barriers or entry portals? The role of cell-cell adhesion during infection. J Cell Biol. 2011;195(3):348-57.
3. Benedicto I, Molina-Jimenez F, Bartosch B, Cosset FL, Lavillette D, Prieto J, et al. The Tight Junction-Associated Protein Occludin Is Required for a Postbinding Step in Hepatitis C Virus Entry and Infection. J Virol. 2009;83(16):8012-20.
4. Lindenbach BD, Rice CM. The ins and outs of hepatitis C virus entry and assembly. Nat Rev Microbiol. 2013;11(10):688-700.
5. Ploss A, Evans MJ, Gaysinskaya VA, Panis M, You H, de Jong YP, et al. Human occludin is a hepatitis C virus entry factor required for infection of mouse cells. Nature. 2009;457(7231):882-6.
6. Bose SK. Hepatitis C virus infection and insulin resistance. World J Diabetes. 2014;5(1):52.
7. Chan SW, Egan PA. Hepatitis C virus envelope proteins regulate CHOP via induction of the unfolded protein response. Faseb J. 2005;19(9):1510-+.
8. Hetz C. The unfolded protein response: controlling cell fate decisions under ER stress and beyond. Nature reviews Molecular cell biology. 2012;13(2):89-102.
9. Mariño G, Niso-Santano M, Baehrecke EH, Kroemer G. Self-consumption: the interplay of autophagy and apoptosis. Nature Reviews Molecular Cell Biology. 2014;15(2):81-94.
10. McGivern DR, Lemon SM. Virus-specific mechanisms of carcinogenesis in hepatitis C virus associated liver cancer. Oncogene. 2011;30(17):1969-83.
11. Pollak M. Insulin and insulin-like growth factor signalling in neoplasia. Nat Rev Cancer. 2008;8(12):915-28.
12. Shackel NA, McGuinness PH, Abbott CA, Gorrell MD, McCaughan GW. Insights into the Pathobiology of Hepatitis C Virus-Associated Cirrhosis: Analysis of Intrahepatic Differential Gene Expression. The American Journal of Pathology. 2002;160(2):641-54.
13. Zhu L-X, Liu J, Xie Y-H, Kong Y-Y, Ye Y, Wang C-L, et al. Expression of hepatitis C virus envelope protein 2 induces apoptosis in cultured mammalian cells. World J Gastroentero. 2004;10(20):2972-8.
14. Ciccaglione AR, Marcantonio C, Tritarelli E, Equestre M, Magurano F, Costantino A, et al. The transmembrane domain of hepatitis C virus E1 glycoprotein induces cell death. Virus Res. 2004;104(1):1-9.
15. Machida K, Cheng KTH, Lai CK, Jeng KS, Sung VMH, Lai MMC. Hepatitis C virus triggers mitochondrial permeability transition with production of reactive oxygen species, leading to DNA damage and STAT3 activation. J Virol. 2006;80(14):7199-207.

16. Li Q, Pène V, Krishnamurthy S, Cha H, Liang TJ. Hepatitis C virus infection activates an innate pathway involving IKK-α in lipogenesis and viral assembly. Nat Med. 2013;19(6):722-9.
17. Israël A. The IKK Complex, a Central Regulator of NF-κB Activation. Cold Spring Harb Perspect Biol. 2010;2(3).
18. Liu Z, Tian Y, Machida K, Lai MMC, Luo G, Foung SKH, et al. Transient activation of the PI3K-AKT pathway by hepatitis C virus to enhance viral entry. J Biol Chem. 2012;287(50):41922-30.
19. Liu S, Yang W, Shen L, Turner JR, Coyne CB, Wang T. Tight Junction Proteins Claudin-1 and Occludin Control Hepatitis C Virus Entry and Are Downregulated during Infection To Prevent Superinfection. J Virol. 2009;83(4):2011-4.
20. Farquhar MJ, Hu K, Harris HJ, Davis C, Brimacombe CL, Fletcher SJ, et al. Hepatitis C Virus Induces CD81 and Claudin-1 Endocytosis. J Virol. 2012;86(8):4305-16.
21. Meertens L, Bertaux C, Dragic T. Hepatitis C Virus Entry Requires a Critical Postinternalization Step and Delivery to Early Endosomes via Clathrin-Coated Vesicles. J Virol. 2006;80(23):11571-8.
22. Blanchard E, Belouzard S, Goueslain L, Wakita T, Dubuisson J, Wychowski C, et al. Hepatitis C Virus Entry Depends on Clathrin-Mediated Endocytosis. J Virol. 2006;80(14):6964-72.
23. Rehermann B, Nascimbeni M. Immunology of hepatitis B virus and hepatitis C virus infection. Nat Rev Immunol. 2005;5(3):215-29.
24. Zona L, Lupberger J, Sidahmed-Adrar N, Thumann C, Harris HJ, Barnes A, et al. HRas signal transduction promotes hepatitis C virus cell entry by triggering assembly of the host tetraspanin receptor complex. Cell Host Microbe. 2013;13(3):302-13.
25. Ito Y, Takeda T, Sakon M, Tsujimoto M, Higashiyama S, Noda K, et al. Expression and clinical significance of erb-B receptor family in hepatocellular carcinoma. Br J Cancer. 2001;84(10):1377-83.
26. Schmitz KJ, Wohlschlaeger J, Lang H, Sotiropoulos GC, Malago M, Steveling K, et al. Activation of the ERK and AKT signalling pathway predicts poor prognosis in hepatocellular carcinoma and ERK activation in cancer tissue is associated with hepatitis C virus infection. Journal of hepatology. 2008;48(1):83-90.
27. Liu XY, Wang TY, Wakita T, Yang W. Systematic identification of microRNA and messenger RNA profiles in hepatitis C virus-infected human hepatoma cells. Virology. 2010;398(1):57-67.
28. Lacalle RA, Mira E, Gomez-Mouton C, Jimenez-Baranda S, Martinez-A C, Manes S. Specific SHP-2 partitioning in raft domains triggers integrin-mediated signaling via Rho activation. J Cell Biol. 2002;157(2):277-89.
