# Supplementary material for: Identification of Entry Factors Involved in Hepatitis C Virus Infection Based on Host-Mimicking Short Linear Motifs
Source: PLoS Comput Biol. 2017 Jan 27;13(1):e1005368. doi: 10.1371/journal.pcbi.1005368 (PMC5302801; doi:10.1371/journal.pcbi.1005368)
Supplement: S3 Table — (PDF) [file pcbi.1005368.s013.pdf]

S3 Table. Published experimental evidence for relations between SLiMs and R6 proteins and between R6 proteins and module functions in Fig 3

**R6 protein**	**Interacting HCV SLiM**	**Description**
**ABL1**	LIG_SH2_STAT5, LIG_SH3_3	Proteins of the ABL tyrosine kinase family, to which ABL1 belongs, regulate *cell-cell adhesion* (module 1)[1]. ABL1 also modulates the organization of *F-actin cytoskeleton* (module 1 and module 7)[1, 2].
**AKT1**	MOD family*	(a) Active AKTs regulate many cellular processes, including *apoptosis* (module 5)[3].<br>(b) HCV E2 induces the phosphorylation of AKT, which enhances HCV entry. This phosphorylation is mediated by the interactions between HCV E2 and two HCV entry host factors, CD81 and CLDN1[4].
**CHUK**	MOD family*	(a) CHUK is a subunit of the IKK complex, which when active can induce the NF-κB pathway to inhibit *apoptosis* (module 5)[5].<br>(b) HCV infection induces CHUK activity[6].
**CSNK2A1, CSNK2A2**	MOD family*	CSNK2A1 and CSNK2A2 are subunits of CK2 protein kinase[7]. CK2 regulates *cell proliferation* (module 4)[8] and *ion channel* activity[9], which is a function associated with module 8 although its enrichment in module 8 was not considered significant (P = 0.055).
**CTTN**	LIG_SH3_3	CTTN regulates *actin cytoskeleton organization* (module 1) by binding and regulating the Arp2/3 complex[10].
**DLG1, DLG4**	LIG_SH3_3	DLG1 and DLG4 are PDZ domain-containing proteins that can regulate *cell-cell junctional signaling* and *organization* (module 6)[11].
**GABARAP**	LIG_LIR_Gen_1, LIG_LIR_Nem_3	GABARAP, a functional homologue of LC3, is a key protein involved in the initial steps of autophagosome formation, which is a critical step of autophagy, a process that regulates *cellular homeostasis* (module 3)[12].
**GRB2**	LIG_SH2_STAT5, LIG_SH3_3	(a) GRB2 plays a role in modulating *actin-based cytoskeleton functions* (module 1 and module 7)[13]. GRB2 is also an adaptor protein that mediates EGFR downstream *signaling* (module 7) and promotes *cell proliferation* (module 4)[14].<br>(b) Silencing of GRB2 expression inhibits HCV entry[15].
**GRK5**	MOD family*	GRK5 is a G–protein-coupled receptor kinase that can enhance the *growth* (module 4) of cancer[16, 17].
**MLLT4**	LIG_FHA_1, LIG_FHA_2	MLLT4 is a cell-cell junction-associated protein, which bridges *Ras signaling toward regulation of cell-cell contact* (module 6)[18].
**NCK1**	LIG_SH2_STAT5, LIG_SH3_3	NCK1 regulates *actin remodeling* (module 1 and module 7) by activating the WASp/Arp2/3 pathway, linking this pathway to *receptor signaling* (module 7)[19] .

S3 Table. Published experimental evidence for relations between SLiMs and R6 proteins and between R6 proteins and module functions in Fig 3 (continued)

R6 protein	Interacting HCV SLiM	Description
**NEDD4**	DOC_WW_Pin1_4	NEDD4 is a ubiquitin ligase that can mediate ubiquitination of the *ion transporter* NHE1[20], a module 8 function although not found to be enriched to a statistical significant level (P = 0.055).
**NEDD4L**	DOC_WW_Pin1_4	NEDD4L is a ubiquitin ligase that can regulate *epithelial sodium transport* (module 8)[21].
**PIK3R1**	LIG_SH2_STAT5	(a) PIK3R1, a regulatory subunit of PI3K, is a pivotal member of the PI3K-AKT *signaling pathway* (module 7)[22] and induces *actin skeleton reorganization* (module 7) in a Cdc42-mediated manner[23].<br>(b) The PI3K-AKT pathway transiently activated by HCV enhances viral entry[4].
**PRKCA**	MOD family*	(a) Activation of PRKCA promotes tumor cell *proliferation* (module 4) and inhibits *apoptosis* (module 5)[24]. Furthermore, PRKCA can promote internalization of integrin β1, which is involved in *integrin signaling* (module 6)[25], via *caveolae-mediated endocytosis* (module 6)[25].<br>(b) PRKCA is a conventional protein kinase C, which plays a role in alpha interferon-mediated HCV clearance[26].
**PRKCD**	MOD family*	PRKCD, a member of the kinase C (PKC) family, can mediate cell *apoptosis* (module 5)[27].
**SRC**	LIG_SH2_STAT5, LIG_SH3_3	(a) SRC modulates *actin cytoskeleton organization* (modules 1 and 7)[28] and the activity of *EGFR* (module 7)[29]. Activation of SRC is regulated by Na/K-ATPase, an *ion transporter* (module 8)[30].<br>(b) The HCV replicon activates SRC by inducing generations of reactive oxygen species[31].
**SHC1**	LIG_SH2_STAT5	(a) SHC1 is an adaptor protein that links EGFR to its downstream *signaling* (module 7)[32].<br>(b) Silencing of SHC1 expression inhibits HCV entry[15].
**TGFBR1**	MOD family*	(a) Formation of the ligand-receptor complex of TGFBR1 and TGF-β activates TGF-β signaling, which is involved in *apoptosis* (module 5)[33].<br>(b) Hepatocytic TGF-β signaling, a tumor-suppression signaling, can be shifted to fibrogenesis by HCV infection and increase risk for HCC[34].

*MOD family: MOD_CK1_1, MOD_CK2_1, MOD_GSK3_1, MOD_NEK2_1, MOD_NEK2_2, and MOD_ProDKin_1.

S3 Table references

1. Zandy NL, Playford M, Pendergast AM. Abl tyrosine kinases regulate cell–cell adhesion through Rho GTPases. PNAS. 2007;104(45):17686-91.
2. Woodring PJ, Litwack ED, O'Leary DDM, Lucero GR, Wang JYJ, Hunter T. Modulation of the F-actin cytoskeleton by c-Abl tyrosine kinase in cell spreading and neurite extension. The Journal of Cell Biology. 2002;156(5):879-92.
3. Song G, Ouyang G, Bao S. The activation of Akt/PKB signaling pathway and cell survival. Journal of Cellular and Molecular Medicine. 2005;9(1):59-71.
4. Liu Z, Tian Y, Machida K, Lai MMC, Luo G, Foung SKH, et al. Transient activation of the PI3K-AKT pathway by hepatitis C virus to enhance viral entry. J Biol Chem. 2012;287(50):41922-30.
5. Israël A. The IKK Complex, a Central Regulator of NF-κB Activation. Cold Spring Harb Perspect Biol. 2010;2(3).
6. Li Q, Pène V, Krishnamurthy S, Cha H, Liang TJ. Hepatitis C virus infection activates an innate pathway involving IKK-α in lipogenesis and viral assembly. Nat Med. 2013;19(6):722-9.
7. Litchfield DW, Bosc DG, Canton DA, Saulnier RB, Vilk G, Zhang CJ. Functional specialization of CK2 isoforms and characterization of isoform-specific binding partners. Mol Cell Biochem. 2001;227(1-2):21-9.
8. Lebrin F, Chambaz EM, Bianchini L. A role for protein kinase CK2 in cell proliferation: evidence using a kinase-inactive mutant of CK2 catalytic subunit alpha. Oncogene. 2001;20(16):2010-22.
9. Bachhuber T, Almaça J, Aldehni F, Mehta A, Amaral MD, Schreiber R, et al. Regulation of the Epithelial Na+ Channel by the Protein Kinase CK2. J Biol Chem. 2008;283(19):13225-32.
10. Cosen-Binker LI, Kapus A. Cortactin: The Gray Eminence of the Cytoskeleton. Physiology. 2006;21(5):352-61.
11. Roberts S, Delury C, Marsh E. The PDZ protein discs-large (DLG): the ‘Jekyll and Hyde’ of the epithelial polarity proteins. FEBS Journal. 2012;279(19):3549-58.
12. Mariño G, Niso-Santano M, Baehrecke EH, Kroemer G. Self-consumption: the interplay of autophagy and apoptosis. Nature Reviews Molecular Cell Biology. 2014;15(2):81-94.
13. Carlier M-F, Nioche P, Broutin-L'Hermite I, Boujemaa R, Clainche CL, Egile C, et al. GRB2 links signaling to actin assembly by enhancing interaction of neural wiskott-aldrich syndrome protein (N-WASp) with actin-related protein (ARP2/3) complex. J Biol Chem. 2000;275(29):21946-52.
14. Downward J. The GRB2/Sem-5 adaptor protein. FEBS Letters. 1994;338(2):113-7.
15. Zona L, Lupberger J, Sidahmed-Adrar N, Thumann C, Harris HJ, Barnes A, et al. HRas

signal transduction promotes hepatitis C virus cell entry by triggering assembly of the host tetraspanin receptor complex. Cell Host Microbe. 2013;13(3):302-13.
16. Kim JI, Chakraborty P, Wang Z, Daaka Y. G-Protein Coupled Receptor Kinase 5 Regulates Prostate Tumor Growth. The Journal of Urology. 2012;187(1):322-9.
17. Kaur G, Kim J, Kaur R, Tan I, Bloch O, Sun MZ, et al. G-protein coupled receptor kinase (GRK)-5 regulates proliferation of glioblastoma-derived stem cells. Journal of Clinical Neuroscience. 2013;20(7):1014-8.
18. Yamamoto T, Harada N, Kano K, Taya S-i, Canaani E, Matsuura Y, et al. The Ras Target AF-6 Interacts with ZO-1 and Serves as a Peripheral Component of Tight Junctions in Epithelial Cells. The Journal of Cell Biology. 1997;139(3):785-95.
19. Chaki SP, Rivera GM. Integration of signaling and cytoskeletal remodeling by Nck in directional cell migration. BioArchitecture. 2013;3(3):57-63.
20. Simonin A, Fuster D. Nedd4-1 and β-Arrestin-1 Are Key Regulators of Na+/H+ Exchanger 1 Ubiquitylation, Endocytosis, and Function. J Biol Chem. 2010;285(49):38293-303.
21. Rotin D, Staub O. Nedd4-2 and the regulation of epithelial sodium transport. Front Physio. 2012;3:212.
22. Miled N, Yan Y, Hon W-C, Perisic O, Zvelebil M, Inbar Y, et al. Mechanism of two classes of cancer mutations in the phosphoinositide 3-kinase catalytic subunit. Science. 2007;317(5835):239-42.
23. Jiménez C, Portela RA, Mellado M, Rodríguez-Frade JM, Collard J, Serrano A, et al. Role of the Pi3k Regulatory Subunit in the Control of Actin Organization and Cell Migration. The Journal of Cell Biology. 2000;151(2):249-62.
24. Michie AM, Nakagawa R. The link between PKC alpha regulation and cellular transformation. Immunol Lett. 2005;96(2):155-62.
25. Caswell PT, Vadrevu S, Norman JC. Integrins: masters and slaves of endocytic transport. Nature Reviews Molecular Cell Biology. 2009;10(12):843-53.
26. Fimia GM, Evangelisti C, Alonzi T, Romani M, Fratini F, Paonessa G, et al. Conventional Protein Kinase C Inhibition Prevents Alpha Interferon-Mediated Hepatitis C Virus Replicon Clearance by Impairing STAT Activation. J Virol. 2004;78(23):12809-16.
27. Reyland ME. Protein kinase Cδ and apoptosis. Biochemical Society Transactions. 2007;35(5):1001-4.
28. Yeatman TJ. A renaissance for SRC. Nat Rev Cancer. 2004;4(6):470-80.
29. Biscardi JS, Maa M-C, Tice DA, Cox ME, Leu T-H, Parsons SJ. c-Src-mediated Phosphorylation of the Epidermal Growth Factor Receptor on Tyr845 and Tyr1101 Is Associated with Modulation of Receptor Function. J Biol Chem. 1999;274(12):8335-43.
30. Weigand KM, Swarts HGP, Fedosova NU, Russel FGM, Koenderink JB. Na,K-ATPase activity modulates Src activation: A role for ATP/ADP ratio. Biochimica et Biophysica Acta (BBA) - Biomembranes. 2012;1818(5):1269-73.

31. Waris G, Turkson J, Hassanein T, Siddiqui A. Hepatitis C virus (HCV) constitutively activates STAT-3 via oxidative stress: Role of STAT-3 in HCV replication. J Virol. 2005;79(3):1569-80.
32. Ravichandran KS. Signaling via Shc family adapter proteins. Oncogene. 2001;20(44):6322-30.
33. Massague J. TGF beta signalling in context. Nat Rev Mol Cell Bio. 2012;13(10):616-30.
34. Matsuzaki K, Murata M, Yoshida K, Sekimoto G, Uemura Y, Sakaida N, et al. Chronic inflammation associated with hepatitis C virus infection perturbs hepatic transforming growth factor β signaling, promoting cirrhosis and hepatocellular carcinoma. Hepatology. 2007;46(1):48-57.
