# Supplementary material for: Identification of Entry Factors Involved in Hepatitis C Virus Infection Based on Host-Mimicking Short Linear Motifs
Source: PLoS Comput Biol. 2017 Jan 27;13(1):e1005368. doi: 10.1371/journal.pcbi.1005368 (PMC5302801; doi:10.1371/journal.pcbi.1005368)
Supplement: S4 Table — (PDF) [file pcbi.1005368.s014.pdf]

S4 Table. Types of PPIs between HCV polyprotein and human protein in PHISTO and their detection methods

PPI	HCV protein	Human protein	Detection method(s)*	PPI type*
1	Polyprotein	FGB	**bimolecular fluorescence complementation**, **two hybrid**, anti tag coimmunoprecipitation, confocal microscopy	Direct
2	Polyprotein	ANXA2	**bimolecular fluorescence complementation**, **electron microscopy**, anti bait coimmunoprecipitation, confocal microscopy	Direct
3	Polyprotein	CD81	**competition binding**, **enzyme linked immunosorbent assay**, **far western blotting**, **fluorescence-activated cell sorting**, **pull down**, other methods	Direct
4	Polyprotein	LTBR	**cosedimentation through density gradient**, **far western blotting**, **pull down**, **two hybrid**	Direct
5	Polyprotein	SCARB1	**cross-linking study**, **fluorescence-activated cell sorting**	Direct
6	Polyprotein	ACTB	**electron microscopy**, anti bait coimmunoprecipitation, confocal microscopy	Direct
7	Polyprotein	RAB18	**electron microscopy**, **pull down**, imaging technique	Direct
8	Polyprotein	GRB2	**enzyme linked immunosorbent assay**, **pull down**, anti bait coimmunoprecipitation	Direct
9	Polyprotein	HCK	**enzyme linked immunosorbent assay**, **protein kinase assay**, **pull down**, anti bait coimmunoprecipitation	Direct
10	Polyprotein	LTF	**far western blotting**, **pull down**, anti bait coimmunoprecipitation, coimmunoprecipitation	Direct
11	Polyprotein	CD209	**fluorescence-activated cell sorting**, **surface plasmon resonance**	Direct
12	Polyprotein	EBAG9	**protein array**	Direct

S4 Table. Types of PPIs between HCV polyprotein and human protein in PHISTO and their detection methods (continued)

PPI	HCV protein	Human protein	Detection method(s)*	PPI type*
13	Polyprotein	CTTN	**protein array**	Direct
14	Polyprotein	CSNK2A2	**protein array**	Direct
15	Polyprotein	HRAS	**protein array**	Direct
16	Polyprotein	CALU	**protein array**	Direct
17	Polyprotein	PDAP1	**protein array**	Direct
18	Polyprotein	RPL29	**protein array**	Direct
19	Polyprotein	PLSCR1	**pull down**, **two hybrid**, anti tag coimmunoprecipitation	Direct
20	Polyprotein	SORBS2	**pull down**, **two hybrid**, other methods, anti tag coimmunoprecipitation	Direct
21	Polyprotein	BIN1	**pull down**, **two hybrid**, other methods, anti bait coimmunoprecipitation, anti tag coimmunoprecipitation, confocal microscopy, tandem affinity purification	Direct
22	Polyprotein	EFEMP1	**pull down**, **two hybrid**, other methods, anti tag coimmunoprecipitation	Direct
23	Polyprotein	VAPA	**pull down**, **two hybrid**, anti tag coimmunoprecipitation, coimmunoprecipitation, confocal microscopy, tandem affinity purification	Direct
24	Polyprotein	NCL	**pull down**, confocal microscopy	Direct
25	Polyprotein	DHCR24	**pull down**	Direct
26	Polyprotein	FAS	**pull down**, anti bait coimmunoprecipitation, confocal microscopy	Direct
27	Polyprotein	TGFBR1	**pull down**, other methods, anti tag coimmunoprecipitation, confocal microscopy	Direct
28	Polyprotein	FN1	**pull down**, **two hybrid**, other methods	Direct
29	Polyprotein	NUCB1	**pull down**, **two hybrid**, other methods	Direct
30	Polyprotein	APOA2	**surface plasmon resonance**, **two hybrid**, other methods	Direct
31	Polyprotein	AHNAK	**two hybrid**	Direct

S4 Table. Types of PPIs between HCV polyprotein and human protein in PHISTO and their detection methods (continued)

PPI	HCV protein	Human protein	Detection method(s)*	PPI type*
32	Polyprotein	AGRN	**two hybrid**	Direct
33	Polyprotein	AXIN1	**two hybrid**, other methods	Direct
34	Polyprotein	LAMA5	**two hybrid**	Direct
35	Polyprotein	SLC31A2	**two hybrid**	Direct
36	Polyprotein	SORBS3	**two hybrid**, other methods	Direct
37	Polyprotein	SRPX2	**two hybrid**	Direct
38	Polyprotein	SLIT3	**two hybrid**	Direct
39	Polyprotein	SPN	**two hybrid**	Direct
40	Polyprotein	RBP1	**two hybrid**	Direct
41	Polyprotein	EFEMP2	**two hybrid**	Direct
42	Polyprotein	VWF	**two hybrid**	Direct
43	Polyprotein	GAPDH	**two hybrid**	Direct
44	Polyprotein	ITGB1	**two hybrid**	Direct
45	Polyprotein	PFN1	**two hybrid**	Direct
46	Polyprotein	COL4A2	**two hybrid**	Direct
47	Polyprotein	VIM	**two hybrid**, confocal microscopy	Direct
48	Polyprotein	C7	**two hybrid**	Direct
49	Polyprotein	VCAN	**two hybrid**	Direct
50	Polyprotein	NID1	**two hybrid**	Direct
51	Polyprotein	PKM2	**two hybrid**, other methods, anti bait coimmunoprecipitation, anti tag coimmunoprecipitation, fluorescence microscopy	Direct
52	Polyprotein	FBLN1	**two hybrid**	Direct
53	Polyprotein	TBXAS1	**two hybrid**, other methods	Direct
54	Polyprotein	GRN	**two hybrid**	Direct
55	Polyprotein	CTGF	**two hybrid**	Direct
56	Polyprotein	FBN1	**two hybrid**	Direct
57	Polyprotein	STAT3	**two hybrid**	Direct
58	Polyprotein	MLLT4	**two hybrid**	Direct
59	Polyprotein	LAMB2	**two hybrid**	Direct
60	Polyprotein	BCAR1	**two hybrid**	Direct
61	Polyprotein	B2M	**two hybrid**, other methods	Direct
62	Polyprotein	FBLN2	**two hybrid**	Direct
63	Polyprotein	MAPK7	**two hybrid**	Direct

S4 Table. Types of PPIs between HCV polyprotein and human protein in PHISTO and their detection methods (continued)

PPI	HCV protein	Human protein	Detection method(s)*	PPI type*
64	Polyprotein	NID2	**two hybrid**	Direct
65	Polyprotein	FBN3	**two hybrid**	Direct
66	Polyprotein	CADPS2	**two hybrid**, other methods	Direct
67	Polyprotein	LTBP4	**two hybrid**	Direct
68	Polyprotein	ALB	**two hybrid**, other methods	Direct
69	Polyprotein	NRP1	**two hybrid**	Direct
70	Polyprotein	PDLIM5	**two hybrid**	Direct
71	Polyprotein	LRRC7	**two hybrid**, other methods	Direct
72	Polyprotein	NRP2	**two hybrid**	Direct
73	Polyprotein	SHARPIN	**two hybrid**, other methods	Direct
74	Polyprotein	FXYD6	**two hybrid**	Direct
75	Polyprotein	MMRN2	**two hybrid**	Direct
76	Polyprotein	PARVG	**two hybrid**, other methods	Direct
77	Polyprotein	SPON1	**two hybrid**	Direct
78	Polyprotein	CELSR2	**two hybrid**	Direct
79	Polyprotein	PNPLA8	**two hybrid**	Direct
80	Polyprotein	PICK1	**two hybrid**	Direct
81	Polyprotein	STAB1	**two hybrid**	Direct
82	Polyprotein	RAI14	**two hybrid**, other methods	Direct
83	Polyprotein	FBLN5	**two hybrid**	Direct
84	Polyprotein	SSX2IP	**two hybrid**	Direct
85	Polyprotein	MAGED1	**two hybrid**, other methods	Direct
86	Polyprotein	LAMC3	**two hybrid**	Direct
87	Polyprotein	PLIN3	**two hybrid**, anti bait coimmunoprecipitation, anti tag coimmunoprecipitation, confocal microscopy	Direct
88	Polyprotein	CD63	**two hybrid**	Direct
89	Polyprotein	FCN2	**two hybrid**	Direct
90	Polyprotein	RBP4	**two hybrid**	Direct
91	Polyprotein	HAMP	**two hybrid**	Direct
92	Polyprotein	PTGS2	**two hybrid**	Direct
93	Polyprotein	CD82	**two hybrid**	Direct
94	Polyprotein	APOA1	**two hybrid**	Direct
95	Polyprotein	APLP2	**two hybrid**	Direct

S4 Table. Types of PPIs between HCV polyprotein and human protein in PHISTO and their detection methods (continued)

PPI	HCV protein	Human protein	Detection method(s)*	PPI type*
96	Polyprotein	FGG	**two hybrid**	Direct
97	Polyprotein	SLC39A1	**two hybrid**	Direct
98	Polyprotein	PPIB	**two hybrid**	Direct
99	Polyprotein	SERPINA1	**two hybrid**	Direct
100	Polyprotein	LRG1	**two hybrid**	Direct
101	Polyprotein	ENO1	**two hybrid**, tandem affinity purification	Direct
102	Polyprotein	EEF1G	**two hybrid**	Direct
103	Polyprotein	SLC4A2	**two hybrid**	Direct
104	Polyprotein	AZGP1	**two hybrid**	Direct
105	Polyprotein	APOB	**two hybrid**, anti bait coimmunoprecipitation	Direct
106	Polyprotein	PDIA6	**two hybrid**	Direct
107	Polyprotein	PON3	**two hybrid**	Direct
108	Polyprotein	FGL1	**two hybrid**	Direct
109	Polyprotein	F2	**two hybrid**	Direct
110	Polyprotein	SEP2	**two hybrid**, anti bait coimmunoprecipitation, anti tag coimmunoprecipitation	Direct
111	Polyprotein	FMNL1	**two hybrid**	Direct
112	Polyprotein	VAPB	**two hybrid**, anti tag coimmunoprecipitation, confocal microscopy, tandem affinity purification	Direct
113	Polyprotein	CDC42BPA	**two hybrid**	Direct
114	Polyprotein	APOH	**two hybrid**	Direct
115	Polyprotein	CTNNB1	**two hybrid**	Direct
116	Polyprotein	NUCB2	**two hybrid**	Direct
117	Polyprotein	RNASET2	**two hybrid**	Direct
118	Polyprotein	PIK3R1	**two hybrid**	Direct
119	Polyprotein	SPTAN1	**two hybrid**	Direct
120	Polyprotein	DST	**two hybrid**	Direct
121	Polyprotein	SCP2	**two hybrid**	Direct
122	Polyprotein	SLMAP	**two hybrid, other methods**	Direct
123	Polyprotein	SERPIND1	**two hybrid**	Direct

S4 Table. Types of PPIs between HCV polyprotein and human protein in PHISTO and their detection methods (continued)

PPI	HCV protein	Human protein	Detection method(s)*	PPI type*
124	Polyprotein	TF	**two hybrid**	Direct
125	Polyprotein	HMGCR	**two hybrid**	Direct
126	Polyprotein	C3	**two hybrid**	Direct
127	Polyprotein	APOE	**two hybrid**	Direct
128	Polyprotein	TNFRSF10B	**two hybrid**	Direct
129	Polyprotein	STX8	**two hybrid**	Direct
130	Polyprotein	AGGF1	**two hybrid**	Direct
131	Polyprotein	MST1	**two hybrid**, other methods	Direct
132	Polyprotein	UTRN	**two hybrid**	Direct
133	Polyprotein	CFH	**two hybrid**	Direct
134	Polyprotein	HRG	**two hybrid**	Direct
135	Polyprotein	APOA5	**two hybrid**	Direct
136	Polyprotein	C4A	**two hybrid**	Direct
137	Polyprotein	SERPING1	other methods	Direct/indirect
138	Polyprotein	VPS35	other methods	Direct/indirect
139	Polyprotein	FLNA	anti bait coimmunoprecipitation, confocal microscopy	Direct/indirect
140	Polyprotein	CANX	anti bait coimmunoprecipitation, confocal microscopy	Direct/indirect
141	Polyprotein	STX12	anti bait coimmunoprecipitation	Direct/indirect
142	Polyprotein	SSB	anti bait coimmunoprecipitation, tandem affinity purification	Direct/indirect
143	Polyprotein	HSP90AA1	anti tag coimmunoprecipitation	Direct/indirect
144	Polyprotein	RAB5A	anti tag coimmunoprecipitation	Direct/indirect
145	Polyprotein	TGFBR2	anti tag coimmunoprecipitation	Direct/indirect
146	Polyprotein	OSBP2	anti tag coimmunoprecipitation, imaging technique	Direct/indirect
147	Polyprotein	TLR2	confocal microscopy	Direct/indirect
148	Polyprotein	C1QBP	confocal microscopy	Direct/indirect
149	Polyprotein	RAB5B	fluorescence microscopy	Direct/indirect
150	Polyprotein	TXN	tandem affinity purification	Direct/indirect
151	Polyprotein	BASP1	tandem affinity purification	Direct/indirect
152	Polyprotein	MARCKS	tandem affinity purification	Direct/indirect
153	Polyprotein	HSPD1	tandem affinity purification	Direct/indirect
154	Polyprotein	HSPA5	tandem affinity purification	Direct/indirect

S4 Table. Types of PPIs between HCV polyprotein and human protein in PHISTO and their detection methods (continued)

PPI	HCV protein	Human protein	Detection method(s)*	PPI type*
155	Polyprotein	MIF	tandem affinity purification	Direct/indirect
156	Polyprotein	TUBB	tandem affinity purification	Direct/indirect
157	Polyprotein	IRS4	tandem affinity purification	Direct/indirect
158	Polyprotein	P4HB	tandem affinity purification	Direct/indirect

*PPIs detected by at least one bold-faced experimental methods are considered “direct”, and “direct/indirect” otherwise. Methods detecting binary interaction, i.e. “direct” PPIs, are: bimolecular fluorescence complementation (26681426), competition binding (23235262), cross-linking study (7708014), electron microscopy (11785754), enzyme linked immunosorbent assay (11906746), far western blotting (18079728), fluorescence-activated cell sorting (11988464), protein array (16629388), protein kinase assay (7708014), pull down (26681426), surface plasmon resonance (7708014), two hybrid (26681426); methods detecting co-complex or non-binary interactions are considered “Direct/indirect” PPIs: anti bait coimmunoprecipitation (18369874), anti tag coimmunoprecipitation (18369874), coimmunoprecipitation (18369874), tandem affinity purification (17640003), cosedimentation through density gradient (7708014), confocal microscopy (17640003), fluorescence microscopy (17640003), imaging technique (17640003), other methods (Not available). Numbers in parentheses are Pubmed ID for the reference that describes the experimental method.
