# Supplementary material for: Identification of Entry Factors Involved in Hepatitis C Virus Infection Based on Host-Mimicking Short Linear Motifs
Source: PLoS Comput Biol. 2017 Jan 27;13(1):e1005368. doi: 10.1371/journal.pcbi.1005368 (PMC5302801; doi:10.1371/journal.pcbi.1005368)
Supplement: S6 Table — (PDF) [file pcbi.1005368.s016.pdf]

S6 Table. KEGG pathways enriched in the six main protein complex groups

KEGG category	ID	Description	P-value of enrichment in complex group						Main functional class		
			A	B	C	D	E	F	Entry	Carcino-genesis	Infectious diseases
4.1 Transport and catabolism	hsa04144	Endocytosis			4.8E–11			3.9E–08			
	hsa04145	Phagosome	5.4E–05								
4.2 Cell motility	hsa04810	Regulation of actin cytoskeleton		2.0E–06				3.8E–12			
4.4 Cellular community	hsa04510	Focal adhesion		1.7E–06		2.9E–06		6.7E–20			
	hsa04520	Adherens junction			7.6E–07	6.5E–09		2.6E–08			
	hsa04530	Tight junction			1.1E–06	8.3E–05					
	hsa04540	Gap junction						1.8E–04			
3.2 Signal transduction	hsa04010	MAPK signaling pathway					8.4E–06	7.7E–05			
	hsa04012	ErbB signaling pathway						1.6E–18			
	hsa04014	Ras signaling pathway						8.1E–12			
	hsa04015	Rap1 signaling pathway			5.3E–08			3.6E–10			
	hsa04020	Calcium signaling pathway						2.1E–04			
	hsa04064	NF–kappa B signaling pathway					8.8E–07				
	hsa04066	HIF–1 signaling pathway						3.5E–07			
	hsa04068	FoxO signaling pathway						3.2E–07			
	hsa04071	Sphingolipid signaling pathway						8.0E–04			
	hsa04072	Phospholipase D signaling pathway						3.3E–11			
	hsa04150	mTOR signaling pathway						2.7E–04			
	hsa04151	PI3K–Akt signaling pathway		1.1E–04				2.7E–09			
	hsa04350	TGF–beta signaling pathway			1.2E–06						
	hsa04390	Hippo signaling pathway			1.5E–10						
	hsa04668	TNF signaling pathway					1.7E–05				
3.3 Signaling molecules and interaction	hsa04512	ECM–receptor interaction		1.7E–06							
4.3 Cell growth and death	hsa04210	Apoptosis					5.2E–05				

S6 Table. KEGG pathways enriched in the six main protein complex groups (continued)

KEGG category	ID	Description	P-value of enrichment in complex group						Main functional class		
			A	B	C	D	E	F	Entry	Carcino-genesis	Infectious diseases
5.1 Immune system	hsa04612	Antigen processing and presentation	3.5E–10								
	hsa04620	Toll–like receptor signaling pathway					1.7E–06				
	hsa04622	RIG–I–like receptor signaling pathway					1.8E–07				
5.2 Endocrine system	hsa04910	Insulin signaling pathway						3.9E–07			
	hsa04915	Estrogen signaling pathway						1.3E–09			
	hsa04917	Prolactin signaling pathway						3.2E–07			
	hsa04920	Adipocytokine signaling pathway					1.7E–05				
6.7 Endocrine and metabolic diseases	hsa04931	Insulin resistance						6.7E–05			
	hsa04933	AGE–RAGE signaling pathway in diabetic complications			5.0E–09						
5.9 Aging	hsa04213	Longevity regulating pathway – multiple species						3.4E–04			
6.1 Cancers: Overview	hsa05200	Pathways in cancer			4.7E–04		8.6E–04	5.1E–10			
	hsa05205	Proteoglycans in cancer		9.9E–05		8.7E–04		6.7E–20			
	hsa05206	MicroRNAs in cancer						2.0E–07			
	hsa05230	Central carbon metabolism in cancer						5.6E–10			
	hsa05231	Choline metabolism in cancer						2.6E–08			
6.9 Infectious diseases: Viral	hsa05160	Hepatitis C					4.2E–05	2.1E–04			
	hsa05161	Hepatitis B					1.0E–05	3.4E–04			
	hsa05168	Herpes simplex infection	8.8E–05				4.0E–05				
6.10 Infectious diseases: Parasitic	hsa05145	Toxoplasmosis					1.9E–04				
