# Supplementary material for: Identification of Entry Factors Involved in Hepatitis C Virus Infection Based on Host-Mimicking Short Linear Motifs
Source: PLoS Comput Biol. 2017 Jan 27;13(1):e1005368. doi: 10.1371/journal.pcbi.1005368 (PMC5302801; doi:10.1371/journal.pcbi.1005368)
Supplement: S1 Appendix — (PDF) [file pcbi.1005368.s017.pdf]

S1 Appendix. Main functionalities associated with KEGG pathways enriched in the HCV-targeted protein complexes.

Fig 4 in the main text shows that the KEGG pathways enriched in VIP-containing protein complexes are associated with three main functions: *entry*, *carcinogenesis*, and *infectious disease*. Below are details of the analysis.

Viral entry

There are five KEGG categories for the enriched pathways ($P < 0.001$; Fig 4 and S6 Table) for viral entry: 1) Cellular community, 2) Transport and catabolism, 3) Cell motility, 4) Signal transduction, and 5) Signaling molecules and interaction. Pathways in these categories are involved in HCV entry into hepatocytes, as described below.

The first entry step involves the attachment of the virus to host cell surface receptors. Many different cell adhesion molecules and their downstream signaling have been reported to be critical for viral entry[1]. In terms of HCV, the tight junction proteins OCLN[2] and CLDN1[3] participate in HCV internalization, and CDH1 (adherens junction protein) and ITGB1 (focal adhesion protein) were recently identified as necessary for virion entry[4, 5]. Consistent with these observations, we identified four enriched KEGG pathways related to cell adhesion (KEGG category: Cellular community; S6 Table): *Tight junction* (enriched in group C and D complexes), *Adherens junction* (enriched in group C, D, and F complexes), *Focal adhesion* (enriched in group B, D, and F complexes), and *Gap junction* (enriched in group F complex). A second key entry step is *Endocytosis* (KEGG category: Transport and catabolism), which involves internalization of the HCV particle[6] and is a pathway enriched in group C and F complexes (S6 Table). We also found that the *Regulation of actin cytoskeleton* pathway (KEGG category: Cell motility) is enriched in group B and F complexes, consistent with the report that the well-studied receptor CD81 can interact with HCV E2 to activate Rho GTPase family proteins (e.g., RAC1 and CDC42); the activated Rho GTPases rearrange actin filaments, allowing the HCV-CD81 complex to relocate so as to interact with tight junction proteins[7].

Several signaling pathways (KEGG category: Signaling transduction) can facilitate HCV entry. For example: 1) during HCV entry, activation of *ErbB signaling* triggers *Ras signaling*, which can induce lateral membrane diffusion of CD81 and the formation of the host entry factor complex[5]; 2) suppression of *MAPK signaling* significantly inhibits HCV entry[5]; 3) activation of *PI3K-Akt signaling* facilitates HCV entry[8]. These signaling pathways are all significantly enriched in the group F complex, indicating that

this group has a strong effect on HCV entry. Finally, the *ECM-receptor interaction* pathway (KEGG category: Signaling molecules and interaction) links the interaction between extracellular proteins and transmembrane proteins, and is enriched in the group B complex.

Carcinogenesis

Eight KEGG pathway categories (Fig 4 and S6 Table) were found to be enriched in complexes associated with these functions: 1) Cancers: Overview, 2) Signaling transduction, 3) Signaling molecules and interaction, 4) Cell growth and death, 5) Ageing, 6) Immune system, 7) Endocrine system, and 8) Endocrine and metabolic diseases. In light of these results it is likely that: 1) HCV E1/E2 can alter cancer-related host functions by targeting VIP-containing targets, suggesting that E1/E2 can modulate hepatocarcinogenesis; 2) E1/E2 may play a role in regulating the responses of host immune system induced by HCV; 3) hepatocellular carcinoma (HCC) progression may be regulated by E1/E2 via targeting the protein complexes that affect hormone-related signaling pathways. Several lines of experimental evidence support these suggestions, as summarized below.

Cancer is a complex disease involving many different signaling pathways, which play important roles in cancer development[9]. We identified several HCC-associated signaling pathways[10], including *MAPK signaling* (enriched in group E and F complexes) and *Ras signaling* (enriched in group F complex) that are involved in cell growth, differentiation and proliferation, and *PI3K-Akt signaling* (enriched in group B and F complexes) and *mTOR signaling* (enriched in group F complex) in cell survival. Furthermore, the inhibition of apoptosis contributes to the development of HCC[11], and *Apoptosis* (KEGG category: Cell growth and death; enriched in group E complex) and its upstream inhibiting pathway *NF-kappa B signaling* (enriched in group E complex) were identified as enriched pathways. Metabolism and glycosylation are also important in caner development[9, 12], and two cancer-associated metabolic pathways, *Central carbon metabolism in cancer* and *Choline metabolism in cancer*, were identified with both enriched in group F complex. Proteoglycans, heavily glycosylated proteins, can affect tumor progression by contributing to proliferation, adhesion, angiogenesis and metastasis of cancer cells[13], and *Proteoglycans in cancer* was identified as an enriched pathway of group B, D, and F complexes. A metabolic disease-associated pathway, *AGE-RAGE signaling pathway in diabetic complications*, is enriched in group C complex; this is a pathway that can be induced by diabetes-associated hyperglycemia[14], can enhance HCC progression[15], and contribute to diabetic complications[14]. Finally, anti-ageing genes may be cancer inhibiting[16] and can be activated by the *Longevity*

regulating pathway, a pathway enriched in group F complex.

HCV infection triggers host innate and adaptive immune responses and induces inflammation[17] although the mechanistic details of the interaction(s) between HCV proteins and host immune systems are not fully understood. S6 Table and Fig 4 show that the *Antigen processing and presentation* pathway is enriched in group A complex, and the *RIG-I-like receptor signaling* and *Toll-like receptor signaling* pathways are enriched in group E complex. These *in silico* results are consistent with experimental data showing that E1/E2 can alter these pathways[18-20] and mediate the escape of HCV from antigen presentation[21]. Insufficient clearance of HCV by the immune system consequently leads to persistent viral infection and chronic inflammation[22], which can result in cirrhosis and HCC[23].

Lastly, our results showed that several hormone signaling and associated disease pathways are significantly enriched in HCV-targeted protein complexes ($P < 0.001$; Fig 4 and S6 Table): *Adipocytokine signaling* in group E, and in group F those relating to the signaling of *insulin*, *estrogen,* and *prolactin*, and the *Insulin resistance* disease pathway. Notably, most of these hormones are associated with regulating HCC progression: For *adipocytokine signaling*, both the promoting[24, 25] and inhibiting effects[26, 27] on HCC progression by leptin and adiponectin have been investigated. Insulin-signaling events are considered to be cancer promoting[28], and estrogen and prolactin are two female hormones that can inhibit HCC progression through their anti-inflammatory effects on hepatocytes[29, 30]. Interestingly, recent studies suggest that HCV infection can induce insulin resistance, which is a cancer risk factor [31] and can repress the host response to anti-viral therapy[32]. Our *in silico* result suggesting the involvement of HCV E1/E2 in insulin resistance is generally in line with the aforementioned reports, but the specifics need to be elucidated experimentally.

Infectious disease

In this third type of KEGG functions, two KEGG infectious pathway categories, Virus and Parasite, were found to be enriched in group A, E and F complexes ($P < 0.001$; Fig 4). Interestingly, not only the *Hepatitis C virus* pathway but also the pathway of *Hepatitis B virus*[33], *Herpes simplex virus*[34], and *Toxoplasma gondi*[35], a pathogen causing *Toxoplasmosis* (see S6 Table), were found to be enriched in group A, E and/or F complexes, indicating that these protein complexes may mediate the interplay between HCV and these other pathogens during coinfection. Indeed, studies have reported that HCV/HBV coinfection occurs frequently and usually results in severer disease progression than infection by HBV or HCV alone[33]. Interestingly, in the group

E complex, six out of eight VIPs in the enriched *Hepatitis B* pathway are also present in the enriched immune-related pathways of *RIG-I-like receptor signaling* and *Toll-like receptor signaling*, suggesting that the immune system may be modulated by the same set of VIPs during HBV/HCV coinfection. However, the cause-and-effect relationship between coinfection and immune modulation is unclear, and further analyses are necessary to elucidate the molecular mechanisms.

S1 Appendix references

1. Bhella D. The role of cellular adhesion molecules in virus attachment and entry. Philosophical Transactions of the Royal Society of London B: Biological Sciences. 2015;370(1661):20140035.
2. Ploss A, Evans MJ, Gaysinskaya VA, Panis M, You H, de Jong YP, et al. Human occludin is a hepatitis C virus entry factor required for infection of mouse cells. Nature. 2009;457(7231):882-6.
3. Evans MJ, von Hahn T, Tscherne DM, Syder AJ, Panis M, Wölk B, et al. Claudin-1 is a hepatitis C virus co-receptor required for a late step in entry. Nature. 2007;446(7137):801-5.
4. Li Q, Zhang Y-Y, Chiu S, Hu Z, Lan K-H, Cha H, et al. Integrative Functional Genomics of Hepatitis C Virus Infection Identifies Host Dependencies in Complete Viral Replication Cycle. PLoS Pathog. 2014;10(5):e1004163.
5. Zona L, Lupberger J, Sidahmed-Adrar N, Thumann C, Harris HJ, Barnes A, et al. HRas signal transduction promotes hepatitis C virus cell entry by triggering assembly of the host tetraspanin receptor complex. Cell Host Microbe. 2013;13(3):302-13.
6. Lindenbach BD, Rice CM. The ins and outs of hepatitis C virus entry and assembly. Nat Rev Microbiol. 2013;11(10):688-700.
7. Brazzoli M, Bianchi A, Filippini S, Weiner A, Zhu Q, Pizza M, et al. CD81 is a central regulator of cellular events required for hepatitis C virus infection of human hepatocytes. J Virol. 2008;82(17):8316-29.
8. Liu Z, Tian Y, Machida K, Lai MMC, Luo G, Foung SKH, et al. Transient activation of the PI3K-AKT pathway by hepatitis C virus to enhance viral entry. J Biol Chem. 2012;287(50):41922-30.
9. Hanahan D, Weinberg RA. Hallmarks of Cancer: The Next Generation. Cell. 2011;144(5):646-74.
10. Whittaker S, Marais R, Zhu AX. The role of signaling pathways in the development and treatment of hepatocellular carcinoma. Oncogene. 2010;29(36):4989-5005.
11. Sun B, Karin M. NF-κB signaling, liver disease and hepatoprotective agents. Oncogene. 2008;27(48):6228-44.
12. Lewis NE, Abdel-Haleem AM. The evolution of genome-scale models of cancer metabolism. Front Physiol. 2013;4:237.
13. Kanehisa M, Goto S. KEGG: Kyoto Encyclopedia of Genes and Genomes. Nucleic Acids Res. 2000;28(1):27-30.
14. Calcutt NA, Cooper ME, Kern TS, Schmidt AM. Therapies for hyperglycaemia-induced diabetic complications: from animal models to

clinical trials. Nat Rev Drug Discov. 2009;8(5):417-29.
15. Yamagishi S-i, Matsui T. Role of receptor for advanced glycation end products (RAGE) in liver disease. European Journal of Medical Research. 2015;20:15.
16. Longo VD, Lieber MR, Vijg J. Turning anti-ageing genes against cancer. Nature Reviews Molecular Cell Biology. 2008;9(11):903-10.
17. Park S-H, Rehermann B. Immune Responses to HCV and Other Hepatitis Viruses. Immunity. 2014;40(1):13-24.
18. Eksioglu EA, Zhu H, Bayouth L, Bess J, Liu H-y, Nelson DR, et al. Characterization of HCV Interactions with Toll-Like Receptors and RIG-I in Liver Cells. PloS one. 2011;6(6):e21186.
19. Foy E, Li K, Sumpter R, Loo Y-M, Johnson CL, Wang C, et al. Control of antiviral defenses through hepatitis C virus disruption of retinoic acid-inducible gene-I signaling. P Natl Acad Sci USA. 2005;102(8):2986-91.
20. Li K, Foy E, Ferreon JC, Nakamura M, Ferreon ACM, Ikeda M, et al. Immune evasion by hepatitis C virus NS3/4A protease-mediated cleavage of the Toll-like receptor 3 adaptor protein TRIF. P Natl Acad Sci USA. 2005;102(8):2992-7.
21. Chang KM, Rehermann B, McHutchison JG, Pasquinelli C, Southwood S, Sette A, et al. Immunological significance of cytotoxic T lymphocyte epitope variants in patients chronically infected by the hepatitis C virus. Journal of Clinical Investigation. 1997;100(9):2376-85.
22. Thimme R, Binder M, Bartenschlager R. Failure of innate and adaptive immune responses in controlling hepatitis C virus infection. FEMS Microbiology Reviews. 2012;36(3):663-83.
23. McGivern DR, Lemon SM. Virus-specific mechanisms of carcinogenesis in hepatitis C virus associated liver cancer. Oncogene. 2011;30(17):1969-83.
24. Chen C, Chang Y-C, Liu C-L, Liu T-P, Chang K-J, Guo I-C. Leptin induces proliferation and anti-apoptosis in human hepatocarcinoma cells by up-regulating cyclin D1 and down-regulating Bax via a Janus kinase 2-linked pathway. Endocr Relat Cancer. 2007;14(2):513-29.
25. Thompson KJ, Lau KN, Johnson S, Martinie JB, Iannitti DA, McKillop IH, et al. Leptin inhibits hepatocellular carcinoma proliferation via p38-MAPK-dependent signalling. HPB. 2011;13(4):225-33.
26. Saxena NK, Fu PP, Nagalingam A, Wang J, Handy J, Cohen C, et al. Adiponectin Modulates C-Jun N-Terminal Kinase and Mammalian Target of Rapamycin and Inhibits Hepatocellular Carcinoma. Gastroenterology. 2010;139(5):1762-73.
27. Chen M-J, Yeh Y-T, Lee K-T, Tsai C-J, Lee H-H, Wang S-N. The promoting

effect of adiponectin in hepatocellular carcinoma. J Surg Oncol. 2012;106(2):181-7.
28. Pollak M. Insulin and insulin-like growth factor signalling in neoplasia. Nat Rev Cancer. 2008;8(12):915-28.
29. Xu H, Wei Y, Zhang Y, Xu Y, Li F, Liu J, et al. Oestrogen attenuates tumour progression in hepatocellular carcinoma. J Pathol. 2012;228(2):216-29.
30. Hartwell HJ, Petrosky KY, Fox JG, Horseman ND, Rogers AB. Prolactin prevents hepatocellular carcinoma by restricting innate immune activation of c-Myc in mice. PNAS. 2014;111(31):11455-60.
31. Djiogue S, Kamdje AHN, Vecchio L, Kipanyula MJ, Farahna M, Aldebasi Y, et al. Insulin resistance and cancer: the role of insulin and IGFs. Endocr Relat Cancer. 2013;20(1):R1-R17.
32. El-Zayadi A-R, Anis M. Hepatitis C virus induced insulin resistance impairs response to anti viral therapy. World J Gastroenterol. 2012;18(3):212-24.
33. Jamma S, Hussain G, Lau DTY. Current Concepts of HBV/HCV Coinfection: Coexistence, but Not Necessarily in Harmony. Curr Hepat Rep. 2010;9(4):260-9.
34. Kaufman B, Gandhi SA, Louie E, Rizzi R, Illei P. Herpes Simplex Virus Hepatitis: Case Report and Review. Clin Infect Dis. 1997;24(3):334-8.
35. Atilla A, Aydin S, Demirdöven AN, Kiliç SS. Severe toxoplasmic hepatitis in an immunocompetent patient. Japanese Journal of Infectious Diseases. 2015;68(5):407-9.
